# Imidazo[1,2-*b*]pyrazole-7-Carboxamide Derivative Induces Differentiation-Coupled Apoptosis of Immature Myeloid Cells Such as Acute Myeloid Leukemia and Myeloid-Derived Suppressor Cells

**DOI:** 10.3390/ijms21145135

**Published:** 2020-07-20

**Authors:** Edit Kotogány, József Á. Balog, Lajos I. Nagy, Róbert Alföldi, Valeria Bertagnolo, Federica Brugnoli, András Demjén, Anita K. Kovács, Péter Batár, Gabriella Mezei, Renáta Szabó, Iván Kanizsai, Csaba Varga, László G. Puskás, Gábor J. Szebeni

**Affiliations:** 1 Laboratory of Functional Genomics, Institute of Genetics, Biological Research Centre, Temesvári krt. 62, H6726 Szeged, Hungary; kotogany.edit@brc.hu (E.K.); balog.jozsef@brc.hu (J.Á.B.); laszlo@avidinbiotech.com (L.G.P.); 2 PhD School in Biology, University of Szeged, H6726 Szeged, Hungary; r.alfoldi@astridbio.com; 3 Avidin Ltd., Alsó kikötő sor 11/D, H6726 Szeged, Hungary; l.nagy@avidinbiotech.com (L.I.N.); a.demjen@avidinbiotech.com (A.D.); a.kovacs@avidinbiotech.com (A.K.K.); i.kanizsai@avidinbiotech.com (I.K.); 4 AstridBio Technologies Ltd., Wimmer Fülöp u. 1, H6728 Szeged, Hungary; 5 Department of Morphology, Surgery and Experimental Medicine, University of Ferrara, Via Fossato di Mortara 70, 44121 Ferrara, Italy; valeria.bertagnolo@unife.it (V.B.); bgf@unife.it (F.B.); 6 Department of Hematology, Faculty of Medicine, University of Debrecen, Nagyerdei körút 98, H4032 Debrecen, Hungary; pbatar@med.unideb.hu (P.B.); gmezei@med.unideb.hu (G.M.); 7 Department of Physiology, Anatomy and Neuroscience, Faculty of Science and Informatics, University of Szeged, Közép fasor 52, H6726 Szeged, Hungary; szaborenata88@gmail.com (R.S.); vacs@bio.u-szeged.hu (C.V.)

**Keywords:** acute myeloid leukemia, myeloid-derived suppressor cells, differentiation, apoptosis

## Abstract

Chemotherapy-induced differentiation of immature myeloid progenitors, such as acute myeloid leukemia (AML) cells or myeloid-derived suppressor cells (MDSCs), has remained a challenge for the clinicians. Testing our imidazo[1,2-*b*]pyrazole-7-carboxamide derivative on HL-60 cells, we obtained ERK phosphorylation as an early survival response to treatment followed by the increase of the percentage of the Bcl-xl^bright^ and pAkt^bright^ cells. Following the induction of Vav1 and the AP-1 complex, a driver of cellular differentiation, FOS, JUN, JUNB, and JUND were elevated on a concentration and time-dependent manner. As a proof of granulocytic differentiation, the cells remained non-adherent, the expression of CD33 decreased; the granularity, CD11b expression, and MPO activity of HL-60 cells increased upon treatment. Finally, viability of HL-60 cells was hampered shown by the depolarization of mitochondria, activation of caspase-3, cleavage of Z-DEVD-aLUC, appearance of the sub-G1 population, and the leakage of the lactate-dehydrogenase into the supernatant. We confirmed the differentiating effect of our drug candidate on human patient-derived AML cells shown by the increase of CD11b and decrease of CD33+, CD7+, CD206+, and CD38^bright^ cells followed apoptosis (IC_50_: 80 nM) after treatment ex vivo. Our compound reduced both CD11b+/Ly6C+ and CD11b+/Ly6G+ splenic MDSCs from the murine 4T1 breast cancer model ex vivo.

## 1. Introduction

Myeloproliferative neoplasms (MPNs) and myelodysplastic syndromes (MDS) are diseases of the hematopoietic stem cells in the bone marrow (BM) where an excess of immature cells is produced, which can frequently evolve to different myeloid leukemias [1,2]. The group of myeloid cells have been determined by the WHO classification, including granulocytic (neutrophil, eosinophil, and basophil), monocytic/macrophage, erythroid, megakaryocytic, and mast cell lineages [1]. The acute myelogenous/myeloid leukemia (AML) is the most common acute leukemia in adults with the incidence of 4.3 in the USA per 100,000 person-years. The AML has the shortest survival among leukemias (in 5 year survival = 24%), especially in the elderly (at age ≥ 65 years) the AML predicts the worst overall median survival (2.67 months) [3]. The AMLs represent a group of heterogeneous forms of myeloid malignancies with diverse genetic abnormalities and different stages of myeloid differentiation. The AMLs originate from myeloid stem cells or myeloid blasts halted in an immature state during hematopoiesis where more than 20% of blasts can be detected in the BM which counteracts with the production of normal blood cells [4].

Current treatment of these myeloid malignancies (MPNs, MDS, and AML) is different. There is no available curative treatment for any type of MPNs. The aims of therapies in MPNs are to limit the severity of symptoms to avoid thrombohemorrhagic complications, and limit anemia and splenomegaly [5,6]. The aims of the therapies in the case of MDS are also to diminish the symptoms, improve the quality of life and decrease progression to AML. Allogeneic stem cell transplantation can be considered under the age of 45 in high risk patients. Supporting cares are blood transfusion and the administration of erythropoietin. Chemotherapy for MDS is performed by the administration of 5-azacytidin, decitabine, and lenalidomide [7]. The treatment of AML mostly relies on high dose chemotherapy. Hematopoietic stem cell transplantation is recommended for younger patients with high risk, therapy refractory or relapsed disease with available HLA-matched donors. The aim of the first line treatment, called induction phase therapy, is to eradicate the leukemic clone and achieve complete remission. The second phase is called consolidation therapy, which helps maintaining complete remission by eliminating minimal residual disease. During induction therapy cytarabine and anthracycline are given, except subtype M3. The overall survival of AML remained poor, however novel therapies have been approved by the FDA, which are CPX-351 (daunorubicin/cytarabine), enasidenib (IDH2 inhibitor), midostaurin (FLT3 inhibitor), and gemtuzumab ozogamicin antibody–drug conjugate (GO) targeting CD33+ cells [8]. The acute promyelocytic leukemia (APL; the AML subtype M3) is treated mainly by all-trans retinoic acid (ATRA) and arsenic trioxide [9,10]. The prototype model used in our study was the human cell line, HL-60 promyeloblasts isolated form a female leukemia patient in 1977 presumed as acute promyelocytic leukemia (APL), but later reclassified to acute myeloblastic leukemia FAB-M2 because of the absence of t(15:17) translocation [11,12,13]. The lineage-uncommitted HL-60 cell line is suitable to model patient-derived samples responsive to differentiation induced chemotherapy. It has been shown that treatment of immature HL-60 cells by ATRA or DMSO resulted in granulocytic differentiation [14,15]. The phorbol ester 12-O-tetradecanoylphorbol 13-acetate (TPA), or 1,25-dihydroxyvitamin D3, induced the differentiation of HL-60 cells into a macrophage like adherent phenotype [16,17]. However, ATRA failed the clinical management of AML lacking the PML–RARα target fusion protein frequent in APL [18,19].

Chemotherapy-induced differentiation has been addressed in the late 1970s and still remained a therapeutic challenge to treat immature myeloid leukemias with high need for novel effective compounds [19]. We have previously synthetized a 67 member imidazo[1,2-*b*]pyrazole-7-carboxamide compound library with potent cytotoxic effect on HL-60 cells [20], and optimization of structure–activity relationship (SAR) resulted in the design of seven more active compounds [21]. These agents induced apoptosis in nanomolar range on HL-60 cells, but the way of action was not revealed. In order to explore the mechanism of action, our imidazo[1,2-*b*]pyrazole-7-carboxamide derivative, published earlier as DU325 [21], was further investigated to elicit its cytotoxic effect on human HL-60 and primary patient-derived AML cells.

Another pathologic condition of myeloid expansion is the ”emergency” granulo-monocytopoiesis in most of the solid malignancies in which, an army of immature myeloid cells leave the bone marrow, called monocytic and granulocytic myeloid-derived suppressor cells (MDSCs) [22]. We have previously reviewed this myeloid infiltrate in solid tumors, how is orchestrated by MDSCs [23]. The murine MDSCs are CD11b^+^ and Gr1^+^ heterogeneous populations of immature myeloid cells developed from bone marrow common myeloid progenitors [24], MDSCs are precursors of granulocytes, monocytes, macrophages, and dendritic cells. MDSCs are classified as Ly6C^+^ monocytic (M-MDSC) and Ly6G^+^ granulocytic (G-MDSC) subpopulations in mice [25]. Due to the lack of Gr1 homologue in humans, the identification of MDSCs is not so evident, human MDSCs consist of phenotypically more heterogeneous population of myeloid cell precursors, briefly M-MDSC (CD11b^+^, HLA-DR^−/low^,CD33^+^, CD14^+^, CD15^−^), G-MDSC (CD11b^+^,HLA-DR^−/low^, CD33^+^, CD15^+^ or CD66b^+^), or the less well defined more immature MDSCs (CD14^−^, CD15^−^) [26,27]. In contrast to AML, MDSCs are not malignant cells, but promote tumor growth by several mechanisms including their inherent immunosuppressive activity, promotion of neoangiogenesis, mediation of epithelial-mesenchymal transition and altering cancer cell metabolism. These protumoral functions of MDSCs offer a myriad of potential anticancer therapeutic targets that we have previously reviewed [28]. We have recently reported the immunomodulatory effect of cisplatin via the suppression of splenic MDSCs in the 4T1 murine breast cancer model [29].

Because MDSCs are immature myeloid cells with immunosuppressive capacity, differentiation into mature myeloid cells, thereby restoration of antitumor T-cell immunity has been proven a promising therapeutic strategy [30,31]. Three agents—the calcitriol receptor agonist 1,25-dihydroxyvitamin D3 (vitamin D3) [32,33], the retinoic acid receptor agonist ATRA [34], and TLR7/8 agonist resiquimod [35]—have been verified to mature MDSCs and reduce tumor nursing condition of immunosuppression [28].

Based on these findings we addressed to investigate the cytotoxic and differentiating effect of our imidazo[1,2-*b*]pyrazole-7-carboxamide derivative on splenic immature myeloid cells, both on M- and G-MDSCs accumulated in 4T1 breast cancer-bearing mice.

## 2. Results

### 2.1. DU325 Drives Survival Pathways as an Early Response to Treatment in HL-60 Cells

**Figure 1:**
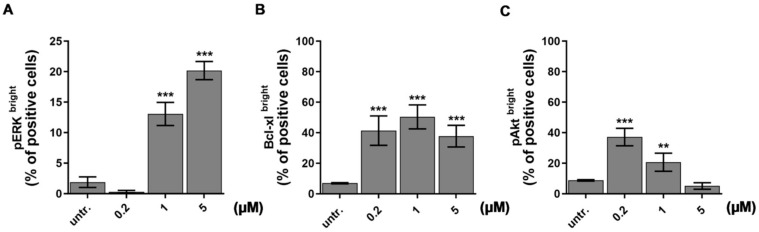
Drug candidate DU325 drives survival pathways as an early response to treatment in HL-60 cells. Using flow cytometry, we obtained ERK phosphorylation (pERK1/2, Thr202/Tyr204) (**A**) as an early response to DU325 stimulation followed by the increase of the percentage of the Bcl-xl (**B**) and pAkt (Ser473) bright cells (**C**). Cells were treated as described in the Materials and Methods Section 4.12.4 for 2 h to assess ERK1/2 phosphorylation, and for 24 h to assess the upregulation of the anti-apoptotic Bcl-xl^bright^ and pAkt^bright^ cells. Data are shown as arithmetic mean values ± standard deviation from triplicate experiments. Statistical significance was calculated in relation to untreated cells and set to ** *p* < 0.01, *** *p* < 0.001.

In order to study the underlying mechanism of action of our recently developed drug candidate, DU325 with anti-leukemic effect [21], the prototype model of human acute myeloblastic leukemia, the HL-60 cells, were treated and assayed. HL-60 cells have been widely used earlier to test differentiation induced chemotherapy [36,37,38]. We have previously observed the asynchronous response of HL-60 cells in terms of morphology treated with DU325 and decided to obtain single cell protein expression data in a subpopulation of cells (bright) by using flow cytometry. We investigated ERK1/2 (extracellular-signal regulated kinases, alternatively MAPK mitogen-activated protein kinases) phosphorylation at Thr202/Tyr204 because ERK has been reported to be involved in myeloid differentiation of HL-60 cells [36,39,40]. The short-term treatment (2 h) of HL-60 cells with 1 μM and 5 μM DU325 induced 13 ± 1.89 % (*p* < 0.001) and 20 ± 1.49 % (*p* < 0.001) of pERK^bright^ positive cells, respectively (Figure 1A and Figure S1A). The accumulation of Bcl-xl has been reported to suppress apoptosis during monocytic differentiation process of HL-60 cells via ERK dependent fashion [41,42]. Indeed, we could detect the increase of the percentage of Bcl-xl^bright^ cells with a peak at 50.3 ± 5.5 % (*p* < 0.001) upon 1 μM DU325 treatment (Figure 1B and Figure S1B). Another factor, the Akt (also known as Protein kinase B or RAC-alpha serine/threonine-protein kinase) that has been reported regulating survival during the differentiation of myeloid cells via NF-κB-dependent induction of Bcl-xl [43]. The percentage of cells with high pAkt (Ser473) was detected 20 ± 4 % (*p* < 0.001) after incubation with 200 nM DU325 (Figure 1C and Figure S1C). The higher concentrations of DU325 caused a decline in the percentage of Bcl-xl^bright^ and pAkt^bright^ cells probably because of the previously reported apoptotic effect [21].

### 2.2. DU325 Induces Differentiation of HL-60 Cells

**Figure 2:**
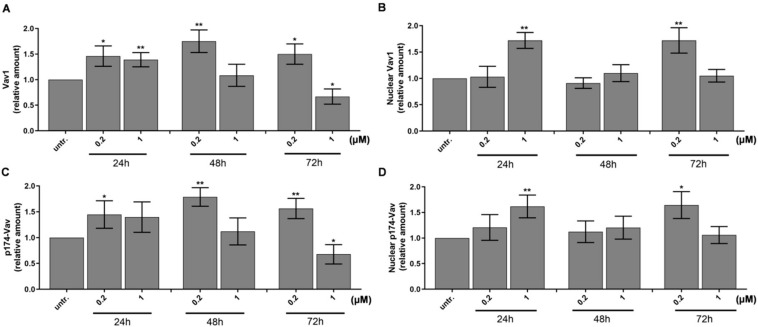
DU325 affects both cellular and nuclear levels of Vav1 in HL-60 cells. Relative levels of Vav1 in whole cells (**A**) and in the nuclei (**B**), and p174-Vav1 (Tyr174) in whole cells (**C**) and in the nuclei (**D**) from HL-60 cells grown in the presence of DU325 at the reported concentrations for the indicated times (h). Cells were assayed as described in the Materials and Methods Section 4.10. The values are deduced from the densitometry of immunochemical bands normalized with β-Tubulin for whole cells or with Lamin B for the nuclei as internal controls of loaded proteins (Figure S2). Data are shown as arithmetic mean values ± standard deviation from triplicate experiments. Statistical significance was calculated in relation to untreated cells and set to * *p* < 0.05, ** *p* < 0.01.

One of the master regulators of myeloid differentiation is Vav1, the hematopoietic cell-specific form of Vav proteins. Vav1 could act via several mechanisms, such as its guanine exchange factor (GEF) activity of GDP/GTP [44], regulating cell motility via cytoskeletal reorganization or modulation of gene expression [45]. The GEF activity of Vav1 is dependent on phosphorylation by either Syk, Zap70, Src, or JNK kinases [37], but Vav1 can have a direct effect on the transcriptional machinery as a component of the transcriptionally active complex or interacting with transcription factors, ribonucleoprotein complexes independent of its GEF activity [46,47,48]. We could detect the accumulation of Vav1 in whole cell lysates (Figure 2A and Figure S2A) and in the nuclei (Figure 2B and Figure S2B) of HL-60 cells with 50% increase treated with 200 nM or 1 μM DU325, respectively, as early as 24 h. Additionally, the increase of the phosphorylation of Vav1 on Tyr-174 residues was detected in the whole cell lysates (Figure 2C) and in the nuclei (Figure 2D).

**Figure 3:**
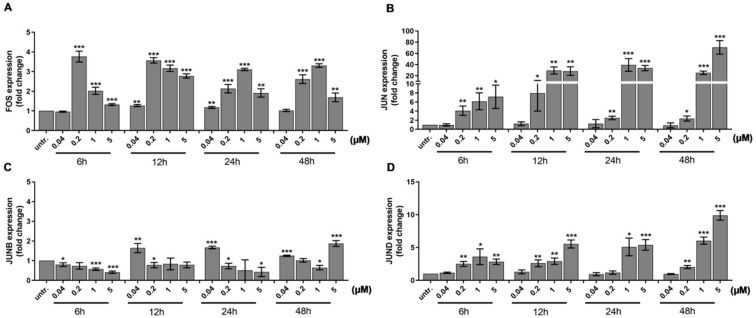
The expression of the members of the AP-1 TF (TF = transcription factor) complex, a driver of cellular differentiation, FOS (**A**), JUN (**B**), JUNB (**C**), and JUND (**D**), was elevated in a concentration and time dependent manner detected by qRT-PCR. Cells were assayed as described in the Materials and Methods Section 4.9. Data are shown as arithmetic mean values ± standard deviation from triplicate experiments. Statistical significance was calculated in relation to untreated cells and set to * *p* < 0.05, ** *p* < 0.01, *** *p* < 0.001.

Mollinedo et al. published that differentiation of HL-60 cells upon stimulation with 1 alpha,25-dihydroxyvitamin D3 required the expression of transcription factor activator protein 1 (AP-1) family members, multiple protein complexes such as FOS and JUN, JUNB, or JUND [49]. The FOS can heterodimerize with JUN members, while the JUN proteins can either homo- or heterodimerize to bind to the target sequences of transcriptionally active DNA elements [50]. We investigated the expression of the AP-1 subunits because both ERK1/2 [51,52] and Vav1 can play a role in the activation of the AP-1 pathway [53,54]. We observed a gradual increase in the expression of FOS (4-times, *p* < 0.001) (Figure 3A), JUN (4-times, *p* < 0.01) (Figure 3B), and JUND (2.5× times, *p* < 0.01) (Figure 3D) after 6 h treatment with 200 nM DU325, while JUNB elevated by 40 nM DU325 after 12 h (1.8× times, *p* < 0.01) (Figure 3C).

**Figure 4:**
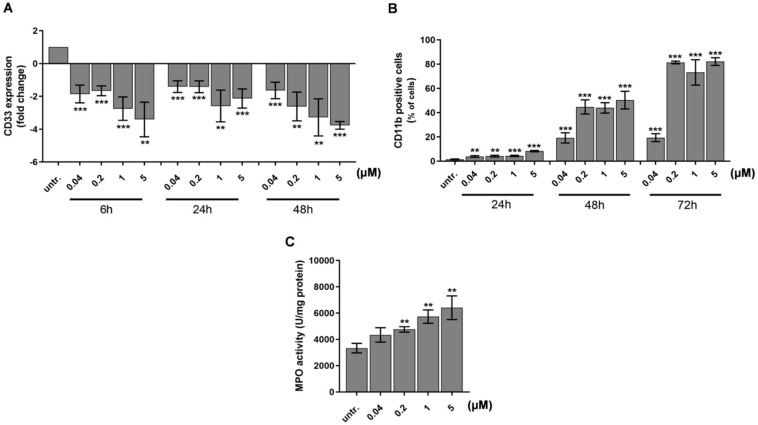
DU325 induces differentiation of HL-60 promyelocytes. As a proof of cellular differentiation, the expression of AML blast and leukemia stem cell marker CD33 decreased (**A**). Matured myeloid cell marker CD11b elevated on the cell surface detected by flow cytometry (**B**). Due to maturation, the myeloperoxidase (MPO) activity of HL-60 cells increased upon treatment after 48 h (**C**). Cells were assayed as described in the Materials and Methods Section 4.12.4. for CD33 and CD11b immunostaining, and 4.11. for the MPO activity. Data are shown as arithmetic mean values ± standard deviation from triplicate experiments. Statistical significance was calculated in relation to untreated cells and set to ** *p* < 0.01, *** *p* < 0.001.

The differentiation of HL-60 immature acute myeloid leukemia cells has been reported with decrease in the expression of early hematopoietic progenitor marker CD33 (Sialic Acid-Binding Ig-Like Lectin 3) and an increase of matured myeloid marker CD11b [55,56]. Next, we investigated the expression of these markers and showed a concentration and time dependent decrease of CD33 as early as 6 h after to 40 nM DU325 treatment with 2-fold decrease (*p* < 0.001) detected by qRT-PCR (Figure 4A). The percentage of CD11b cells was followed by flow cytometry in order to monitor the size and granularity of the cells at single cell resolution. The induction of CD11b positive cells proportional to the maturation of HL-60 started from 5% (24 h to 40 nM DU325, *p* < 0.01) and reached a plateau at 90% (72 h 200 nM DU325, *p* < 0.001) (Figure 4B and Figure S3). Next, we followed the fate of HL-60 cells, whether the differentiation leads to granulocytic or monocytic direction. The FSC-SSC (FSC = Forward scatter, SSC = Side scatter) dot plots showing increasing granularity supported the granulocytic differentiation (Figure S3). In order to clarify the result of maturation MPO (myeloperoxidase) activity, a marker of neutrophil granulocytes was measured after 48 h treatment with DU325. The differentiating agent DU325 at 200 nM resulted in the elevation of MPO activity 4757 ± 205 vs. (versus) untreated 3335 ± 363 U/mg protein (*p* < 0.01) with a maximum of 6405 ± 901 U/mg protein (5 μM) (*p* < 0.01) (Figure 4C).

### 2.3. Differentiation of HL-60 Cells Is Followed by Apoptotic Cell Death

**Figure 5:**
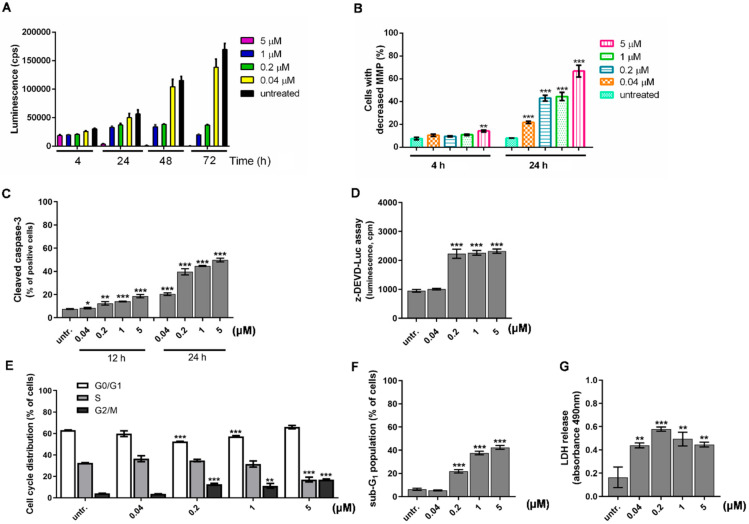
Differentiation of promyelocytic leukemia cells is followed by apoptotic cell death. Differentiation of HL-60 cells was accompanied by cell death detected by RealTime-Glo^TM^ MT Cell Viability Assay (**A**). The depolarization of the mitochondrial membrane potential (MMP) (**B**), activation of caspase-3 (**C**), and the cleavage of Z-DEVD-aLuc (**D**) were observed after 24 h. Cell cycle was arrested in G2/M detected after 72 h incubation (**E**). Finally, as a proof of massive cell death, we showed both the appearance of the hypodiploid apoptotic cells in the sub-G1 population (**F**) and the leakage of the lactate-dehydrogenase (LDH) into the supernatant (**G**) after 72 h incubation. Data are shown as arithmetic mean values ± standard deviation from triplicate experiments. Statistical significance was calculated in relation to untreated cells and set to * *p* < 0.05, ** *p* < 0.01, *** *p* < 0.001.

The cytotoxic effect of DU325 on HL-60 cells was published with IC_50_: 66 nM (half maximal inhibitory concentration) and the effect relied on apoptosis not necrosis confirmed by the exclusive AnnexinV/propidium iodide staining [21]. Since Doyle at al. published earlier that induction of differentiation of HL-60 cells is accompanied by apoptotic cell death [57], we further elucidated the effect of DU325 on the viability of HL-60 cells. The RealTime-Glo™ MT Cell Viability Assay detects the reduction of Nanoluc^®^ substrate by metabolic active cells [58], it revealed that albeit DU325 negatively affects cell viability, this is an asynchronous process not causing a simultaneous cell death in all cells (Figure 5A). The luminescent signals (cps = count per seconds) of metabolically active cells to 1 μM DU325 treatment were 3.31 × 10^4^ vs. 5.27 × 10^4^ untr. (untreated) after 24 h (*p* < 0.001); 3.4 × 10^4^ vs. 1.114 × 10^5^ untr. after 48 h (*p* < 0.001); 2 × 10^4^ vs. 1.64 × 10^5^ untr. after 72 h (*p* < 0.001). The decrease of the mitochondrial membrane potential (MMP) was measured by the JC-1 assay. The early treatment with 5 μM DU325 induced a moderate depolarization of the mitochondria in 14.2 ± 0.9 % of cells vs. in 7.5 ± 1.1 untreated (steady state) cells after 4 h (*p* < 0.01) (Figure 5B and Figure S4). The disturbance of the mitochondrial homeostasis was shown after 24 h by the increase of cells with depolarized mitochondria in 21, 43, 44, and 66% of the cells treated with 40 nM, 200 nM, 1 μM, and 5 μM DU325, respectively (*p* < 0.001) (Figure 5B and Figure S4). Next, we studied that the cell death following the differentiation of HL-60 cells is caspase-dependent or independent. We assayed the percentages of cells bearing cleaved active caspase-3 by flow cytometry and showed a gradual increase on a dose and time dependent manner (Figure 5C and Figure S5). After 24 h of administration of 1 μM or 5 μM DU325 induced the percentage of active caspase-3 positive cells at peaks 44.6 ± 0.23 (*p* < 0.001) or 49.7 ± 1.13 (*p* < 0.001) vs. untreated 7.5 ± 0.21. The substrate of caspase-3, Z-DEVD-aLuc was cleaved by the lysates of HL-60 cells treated with 200 nM DU325 measured as 2227 ±156 vs. 948 ± 45 cps untr. (*p* < 0.001) after 24 h (Figure 5D). The distribution of cell cycle phases and the percentages of hypodiploid cells, as a final stage of apoptosis with the internucleosomal degradation of the DNA was measured by flow cytometry. The ModFit software was used for gating on the intact cells in the FL2-A/FL2-W dot plots and for the quantitation of the percentages of cells in different cell cycle phases. Our drug candidate led to the accumulation of cells in G2/M at the expense of G0/G1 in the case of 200 nM (12.6 ± 0.67 %, *p* < 0.001) and 1 μM (11.2 ± 1.52 %, *p* < 0.001) treatment vs. untreated (4.2 ± 0.05 %) (Figure 5E and Figure S6). The highest concentration applied, 5 μM DU325 reduced the percentages of cells in the S-phase (17.06 ± 1.59 vs. 32.5 ± 0.13 untreated, *p* < 0.001) parallel with the accumulation of G2/M (16.9 ± 0.49 vs. 4.2 ± 0.05 untreated, *p* < 0.001) (Figure 5D). The sub-G1 population with degraded DNA, as a final apoptotic step, increased in a concentration and time dependent manner with 21.97 ± 0.97 % (200 nM, *p* < 0.001), 37.63 ± 1.04 % (1 μM, *p* < 0.001), 42.3 ± 1.13 % (5 μM, *p* < 0.001) vs. untreated (6.2 ± 0.66 %), respectively (Figure 5F). Finally, the leakage of the LDH (lactate dehydrogenase) into the supernatant was measured as a sign of compromised plasma membrane integrity of late apoptotic cells [59]. We could detect LDH in the media even after treatment with 40 nM DU325 (2.6-fold increase, *p* < 0.01) for 72 h (Figure 5G).

### 2.4. DU325 Induces Differentiation and Apoptosis of Human Primary AML Cells

Although HL-60 cells serve as a popular model of human AML in thousands of publications until now, these cells bear the limitations of cell line models such as lab to lab variations caused by genetic drift (HL-60 cell are cultured ex vivo from 1977), possible infection with Mycoplasma and maximum representing only one, the host patient’s condition. In order to validate the differentiating effect of our imidazo[1,2-*b*]pyrazole-7-carboxamide derivative on AML cells, human primary bone marrow aspirate cells were treated from patients diagnosed with AML. Here, we addressed to demonstrate two case studies of human AML cells treated ex vivo, because both the pharmacodynamics and pharmacokinetics of DU325 should be clarified during future pre-clinical studies to design in vivo experiments.

**Figure 6:**
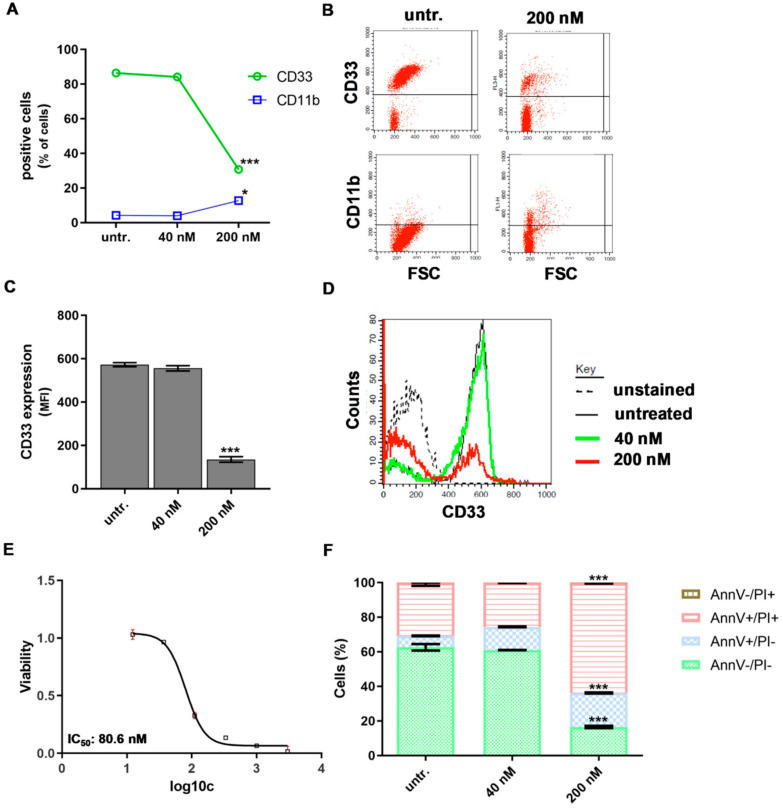
The human ‘AML1’ patient-derived bone marrow aspirate cells responded to DU325 with differentiation-coupled apoptosis. The loss of the percentage of CD33 positive and an increase of CD11b positive cells were assayed by flow cytometry (**A**) and (**B**) after 48 incubation with 200 nM DU325. The median fluorescent intensity (MFI) proportional with CD33 expression on the cells also decreased by 200 nM DU325 (**C**) and (**D**). The resazurin viability assay determined IC_50_ value of DU325 on the primary human patient-derived ‘AML1’ cells after 72 h treatment of 80.6 nM. Concentrations of DU325 were as follows, 3 μM, 1μM, 333 nM, 111 nM, 37 nM, and 12 nM (**E**). The combined Annexin V and propidium iodide (AnnV/PI) flow cytometry staining showed an increase of both AnnV+/PI- early and AnnV+/PI+ late apoptotic cells after treatment with 200 nM DU325 for 48 h (**F**). Data are shown as arithmetic mean values ± standard deviation. Statistical significance was set to *** *p* < 0.001.

The percentage of CD33 positive human ‘AML1’ patient-derived bone marrow aspirate cells dropped from 86% to 30.6 ± 0.7, (*p* < 0.001) and the percentage of CD11b cells increased from 4% to 12.6 ± 1.4, *p* < 0.05) upon 200 nM DU treatment for 48 h (Figure 6A,B).

The density of CD33 marker on the ‘AML1’ cells showed 65% reduction (*p* < 0.001) after treatment with DU325 detected by flow cytometry (Figure 6C,D). Similarly to the HL-60 cell line, the viability of human primary ‘AML1’ cells was hampered by DU325, with 80.6 nM IC_50_ value (Figure 6E). Differentiation-coupled apoptosis was detected by AnnV/PI (Annexin V/propidium iodide) flow cytometry staining showing 63.5 ± 0.28 % late apoptotic AnnV+/PI+ cells vs. 16.4 ± 0.62 % untreated after 200 nM DU325 administration for 48 h (Figure 6F).

**Figure 7:**
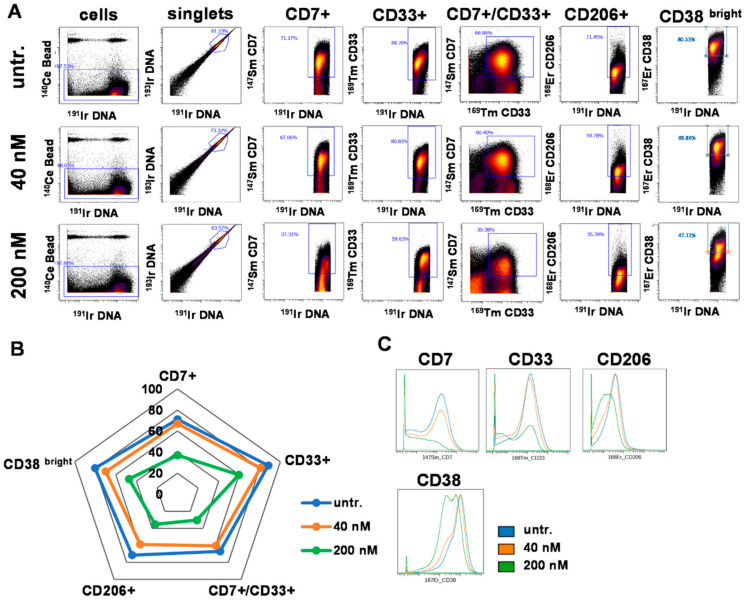
The percentage of CD7+, CD33+, CD7+/CD33+, CD206+, and CD38^bright^ human AML2, patient-derived bone marrow aspirate cells decreased after the treatment with DU325 ex vivo. Mass cytometry was used for multiparametric immunophenotyping of AML2 patient-derived cells treated by DU325 for 48 h. Gating strategy is presented to define single cells and sub-populations of AML cells (**A**). Gating strategy for 11 additional subpopulations can be found in Figure S8. The pentagram of the population trajectories (**B**) shows the decrease of the percentages of CD7+, CD33+, CD7+/CD33+, CD206+, and CD38^bright^ AML2 cells treated with 40 nM (orange) or 200 nM DU325 (green) ex vivo for 48 h. The overlay of the histograms demonstrates the lower expression of CD7, CD33, CD206, and CD38 followed by DU325 treatment in nanomolar range (**C**).

In order to further explore the effect of DU325, cells of another case of human primary AML, the ‘AML2’ were treated and analyzed by single cell resolution mass cytometry. The multiparametric immunophenotyping was carried out by the simultaneous labeling with fifteen metal-tag labeled antibodies. The number of singlets (single cell events) analyzed by CyTOF (cytometry by time-of-flight) were as follows, 380,906 for untreated; 358,557 for 40 nM DU325 treated; and 309,345 for 200 nM DU325 treated. The viability of ‘AML2’ cells was assessed by the uptake of ^195^Pt cisplatin analyzed by CyTOF. The imidazo[1,2-*b*]pyrazole-7-carboxamide derivative, DU325 reduced the viability of patient derived ‘AML2’ cells from 86.27 to 62.18% applied at 200 nM for 48 h (Figure S7). Markers of AML such as CD7 (T-cell Leukemia Antigen), CD33, CD206 (mannose receptor C type 1, MRC1), and CD38 (ADP-Ribosyl Cyclase 1) were included in the antibody panel. The percentage of these subpopulations was determined by manual gating (Figure 7A) and visualized on a radar plot (Figure 7B). The most potent effect of DU325 was at 200 nM decreasing the following subpopulations of CD7+ cells from 71.17 to 37.31%, CD33+ cells from 88.29 to 59.63%, CD7+/CD33+ cells from 66.9 to 30.38%, CD206+ cells from 71.45 to 35.39%, and CD38^bright^ 80.33 to 47.12% (Figure 7A,B). The trajectories of the radar plot delineate the expression profile of the markers of the patient-derived AML2 cells treated by 40 nM, 200 nM DU325 or left untreated. The percentage of the AML2 cells gated by the following markers; CD45, CD11b, CD38 (all), CD163, CD11c, HLA-DR, CD16, CD19, CD66, CD14, CD3, and CD36 upon 40 nM or 200 nM DU325 treatment is shown in Figure S8. The median metal intensity values proportional with the expression intensities of the proteins on the singlets were as follows; for CD7: 91.69, 75.82, and 12.08; CD33: 123.69, 93.09, and 36; CD206: 33.35, 26, and 13.24, CD38: 843.14, 655.18, and 306.7 as untreated, 40 nM or 200 nM DU325 treated, respectively (Figure 7C). Taken together, we could detect at single cell resolution by mass cytometry that both CD7+, CD33+, CD7+/CD33+, CD206+, and CD38^bright^ human primary bone marrow aspirate cells were the most sensitive to the ex vivo treatment with our drug candidate DU325.

**Figure 8:**
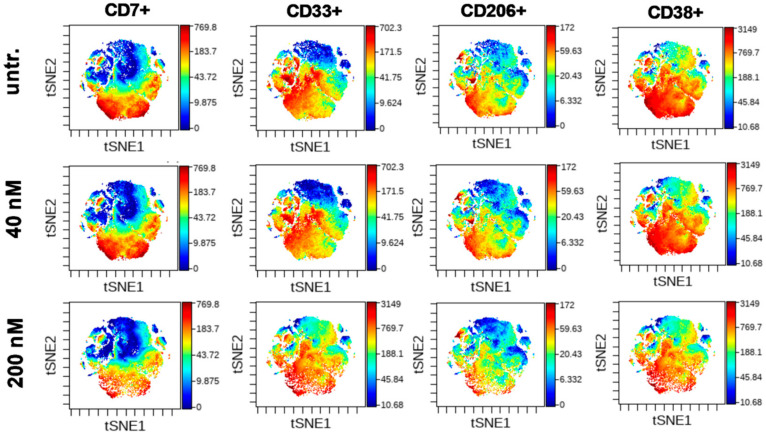
The most sensitive cells to DU325 treatment were CD7+, CD33+, CD206+, and CD38 ^bright^. The multidimensional viSNE plots of mass cytometry experiments illustrate the expression intensity of CD7, CD33, CD206, and CD38 markers showing a partial overlay, common marker expression of these subpopulations of human AML2 patient-derived cells. The coloration is proportional to the expression intensity (blue = low, red = high). The AML2 cells were treated with 40 nM or 200 nM DU325 ex vivo for 48 h. The viSNE plots of the rest, the other 11 markers can be found in Figure S9, the list of all antibodies used for mass cytometry can be found in Section 4.13.

The t-distributed stochastic neighbor embedding (tSNE) algorithm enables the multidimensional analysis of the cells simultaneously taking into account all of fifteen antibodies in the antibody panel for each event at single cell resolution. The cell relatedness of cells with common marker expression is shown by the proximity of these cells in the islands of the visualization of stochastic neighbor embedding (viSNE) graphs. The coloration is proportional to the expression intensity (blue = low, red = high) for each marker separately [60]. This unsupervised analysis verified that CD7+, CD33+, CD206+, and CD38 ^bright^ cells were the most sensitive to DU325 and these cells represent partially overlapping subpopulations of AML2 human primary bone marrow aspirate cells (Figure 8). The tSNE analysis of the other eleven markers of the antibody panel showed strong HLA-DR expression, CD45 dim positivity, and weak CD11c+ intensity, with almost the absence of CD163+, CD11b+, CD66+, CD36+, CD16+, CD14+, CD19+, and CD3+ populations (Figure S9).

### 2.5. DU325 Treatment Ex Vivo Reduced Both Monocytic and Granulocytic Splenic MDSCs from the 4T1 Murine Breast Cancer Model

**Figure 9:**
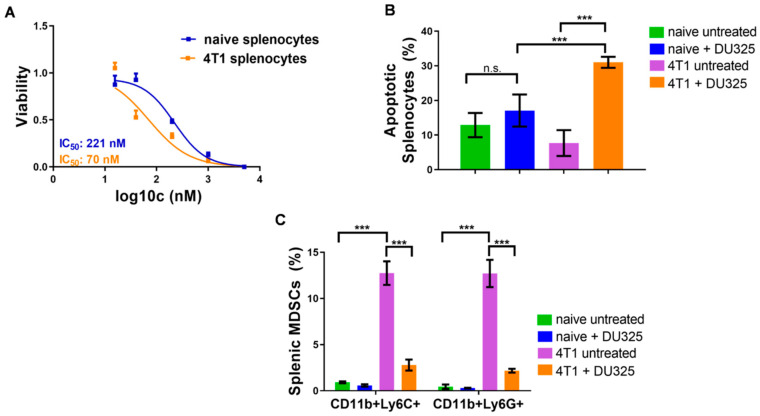
DU325 treatment hampered the viability of splenocytes, both CD11b+/Ly6C+ and CD11b+/Ly6G+ splenic MDSCs. Cells were treated with 5 μM, 1 μM, 200 nM, 40 nM, and 20 nM DU325 for 72 h and assayed by the resazurin viability test (**A**). The percentage of AnnV+/PI+ late apoptotic cells was increased after treatment with 200 nM DU325 for 72 h (**B**). The immunofluorescent staining showed a reduction of both monocytic CD11b+/Ly6C+ and granulocytic CD11b+Ly6G+ MDSCs treated with 200 nM DU325 for 72 h ex vivo (**C**). Data are shown as arithmetic mean values ± standard deviation from triplicate experiments. Statistical significance was set to *** *p* < 0.001.

We have previously reviewed the contribution of MDSCs to the tumor microevolution [23,28] and showed the expansion of these immature myeloid cells in the spleen of 4T1 breast cancer bearing mice [29]. Here, we addressed to investigate the effect of DU325 treatment on the viability of these MDSCs ex vivo. The resazurin viability assay showed three times sensitivity of splenocytes from 4T1 tumor bearing mice with 70.64 nM IC_50_ value compared to naive cells (IC_50_: 221.9 nM) (Figure 9A). The percentage of late apoptotic (AnnV+/PI+) cells from tumor-bearing mice increased upon DU325 treatment to 31 ± 1.6% vs. 7.6 ± 3.7% (*p* < 0.001) untreated, and it was a significant increase also compared to DU325 treated naive splenocytes with 17.08 ± 4.6% (*p* < 0.001) (Figure 9B). After the incubation with DU325 both monocytic CD11b+/Ly6C+ and granulocytic CD11b+/Ly6G MDSCs were reduced to six times less percentage (*p* < 0.001). The gating strategy for monocytic CD11b+/Ly6C+ and for granulocytic CD11b+/Ly6G MDSCs can be found in Figure S10 and in Figure S11, respectively.

## 3. Discussion

Multi-target drugs changing cancer cell homeostasis via modulating apoptotic, differentiation, and metabolic pathways have been applied to treat different malignancies [61]. However, novel agents acting via different mechanisms were approved by the FDA to manage AML and have been recently reviewed [8,62], the overall survival of AML remained poor, especially in the elderly. Another pathophysiologic condition is the expansion of immature myeloid cells, such as MDSCs with potent immunosuppression in solid malignancies. We have previously reviewed the inventory of drugs targeting these MDSCs and discussed differentiation induction therapy as an already proved concept by the administration of vitamin D3 or ATRA [28]. However, reducing MDSCs as a part of supportive immunotherapy still is not implemented in the clinical routine. Along SAR optimization we have designed and synthetized a 74 member library of imidazo[1,2-*b*]pyrazole-7-carboxamides with apoptotic effect on HL-60 cells [20,21]. In order to elucidate the mechanism of action and target immature myeloid cells of AML or MDSCs, our lead imidazo[1,2-*b*]pyrazole-7-carboxamide derivative, DU325 was tested on HL-60 or human primary AML bone marrow aspirate cells, or on the viability of murine 4T1 breast cancer-derived splenic MDSCs ex vivo.

**Figure 10:**
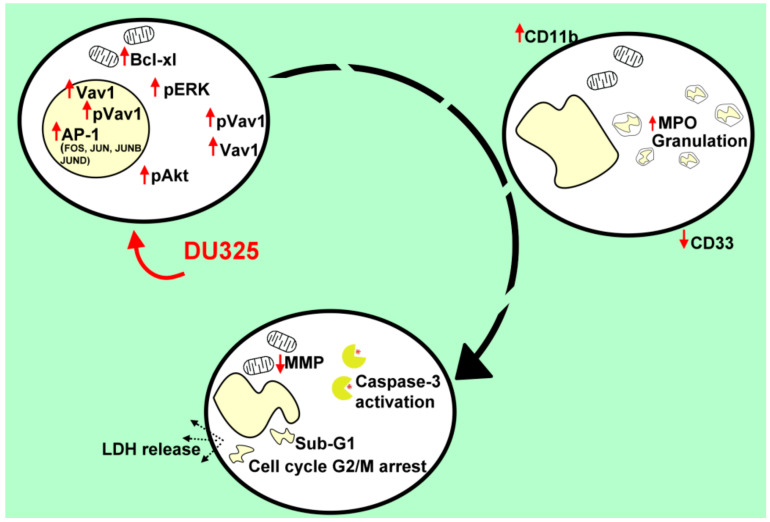
The series of events followed after the treatment of HL-60 cells with DU325. We obtained ERK phosphorylation as an early survival response to treatment followed by the increase of the percentage of the Bcl-xl^bright^ and pAkt^bright^ cells. The induction of Vav1, pVav1, and the AP-1 complex is a driver of cellular differentiation; FOS, JUN, JUNB, and JUND were elevated on a concentration and time-dependent manner. As a proof of granulocytic differentiation, the cells remained nonadherent and the expression of CD33 decreased; the granularity, CD11b expression, and MPO activity of HL-60 cells increased upon treatment. Finally, viability of HL-60 cells was hampered shown by the depolarization of mitochondria, activation of caspase-3, G2/M cell cycle arrest, appearance of the sub-G1 population, and the leakage of the lactate-dehydrogenase into the supernatant.

Using the HL-60 model cell line, we showed that DU325 drives early survival signals. The percentage of pERK^bright^ positive cells (phosphorylation of ERK1/2 at Thr202/Tyr204) was increased after 2 h of treatment with DU325 (Figure 10). In agreement with this, the involvement of ERK1/2 phosphorylation in cell growth and myeloid differentiation was previously published by others upon administration of ATRA [36], G-CSF and M-CSF [39], or Xenospontine [40]. There was an increase in the percentage of antiapoptotic Bcl-xl^bright^ cells in line with previous reports showing ERK dependent survival of Bcl-xl^bright^ HL-60 cells [41,42]. The inhibition of apoptosis or the early cell survival was accompanied with the increase of pAkt^bright^ (Ser473) HL-60 cells after DU325 treatment, which is known to contribute to the NF-κB-driven induction of Bcl-xl [43]. Next, we investigated the accumulation of the hematopoietic cell specific Vav1 protein, one of the master regulators of myeloid leukemia cell differentiation [37]. The Vav1 coordinates cellular maturation via its versatile functions such as GEF activity, affecting cell motility, and morphology via influencing reorganization of actin cytoskeleton and modulating gene expression via interacting with transcription factors, ribonucleoprotein complexes [45,63]. We showed the accumulation of both Vav1 and p174-Vav1 (Tyr174) in the cytoplasm and nuclei and that was associated with changes in gene expression of AP-1 TF complex leading to increased granularity and loss of CD33 expression. It has been known that both ERK1/2 [51,52], and Vav1 can affect AP-1 expression [53,54]. The expression of the members of AP-1 TF complex: FOS, JUN, JUNB, and JUND were gradually increased after DU325 stimulation. The expression of other transcription factors was investigated such as Spl1 (Transcription factor PU.1), RUNX1 (Runt-related transcription factor 1), CEBPA (CCAAT/enhancer binding protein alpha), GABP1 (GA-binding protein subunit beta-1), and IRF1 (Interferon regulatory factor-1), but these were not differentially expressed upon DU325 treatment. Brugnoli et al. found that Vav1 can be recruited to the promoter to drive the expression of CD11b [48], and both the members of AP-1 TF may play in the subsequent steps driving cellular differentiation. However, we did not addressed chromatin immunoprecipitation (ChIP) to isolate target genes of AP-1 TF or Vav1 ribonucleoprotein complexes, as we focused on the interrogation of DU325 caused phenotypic changes at single cell level. Differentiated HL-60 cells increased their granularity detected on SSC-FSC dot plots, remained nonadherent and short lived, decreased CD33 and increased CD11b level and MPO activity suggesting granulocytic differentiation. The HL-60 cells poorly, only in 5% express CD34 that is why it was not monitored [64]. The proposed role of the survival pathways (ERK phosphorylation, Bcl-xl induction, and Akt phosphorylation) in the induction of Vav1 and AP-1 is the guidance and orchestration of cellular differentiation of the studied immature myeloid cells (Figure 10) [65,66].

Finally, differentiation-induced apoptosis was assayed on HL-60 cells upon DU325 administration because it was previously published that maturation of HL-60 cells spontaneously leads to apoptosis in these cells [55,57,67]. Maianski et al. published that the major role of the mitochondria of the matured neutrophils is to drive the apoptotic cell death [68]. Indeed, we showed the loss of viability in a real-time assay and the depolarization of the mitochondria driving the intrinsic pathway of apoptosis. Additionally, we showed the subsequent steps of apoptosis, activation of caspase-3, cleavage of Z-DEVD-aLuc, increase of hypodiploid sub-G1 population, and leakage of LDH into the supernatant after 72 h of treatment (Figure 10). The effect of DU325 was dissected in HL-60 cells bearing c-myc proto-oncogene amplification as a canonic model of human AML cells [11].

We have further investigated human primary bone marrow aspirate cells form different AML patients, as two different case studies, because the AML sub-types greatly vary regarding the genetic mutations. The treatment of primary AML cells was performed ex vivo. However, we could show the loss of CD33 and increase of CD11b on AML1 cells followed by apoptosis with 80.6 nM IC_50_ value. The response of patient-derived AML2 cells was monitored by that state-of-the-art single cell mass cytometry providing multiplex immunophenotyping and complex bioinformatic analysis. Performing immunostaining with 15 antibodies revealed the most sensitive subpopulations to DU325 treatment, namely, CD7+, CD33+, CD7+/CD33+, CD206+, and CD38^bright^ shown by manual gating and unsupervised viSNE analysis. The CD7, CD33, and CD38 are well-known therapeutic targets of AML [69,70,71]. Although the mannose receptor C type 1 (MRC1), the CD206 has been described as a marker of M2, alternatively polarized macrophages or M2-like monocytes with the co-expression of CD14 and CD163 [23], we detected CD206 cells distinct from CD14+ and CD163+ cells as it shown in the viSNE maps. Using single cell RNA sequencing and Cancer Genome Atlas data of 179 AML patients, Galen et al. found that higher expression of CD206+ was correlated with poor survival offering CD206 as a good target in AML [72].

Based on our results and on the fact that differentiation of immature APL or AML cells increase the sensitivity to chemotherapeutic drugs [73,74] novel studies should be initiated to examine the efficacy of the combination of differentiating agents with existing anticancer drugs.

Another pathologic condition is the expansion of immature myeloid cells, such as MDSCs in the bone marrow and tumor microenvironment of solid malignancies [75]. We have previously reviewed the pharmaceutical drugs or therapeutic efforts for the selective ablation of these MDSCs, inhibiting their recruitment or limiting their functions [28]. Using our imidazo[1,2-*b*]pyrazole-7-carboxamide we could induce the apoptosis of both CD11b+/Ly6C+ monocytic and CD11b/Ly6G+ granulocytic MDSCs isolated from the spleen of 4T1 tumor bearing mice. Maturation of MDSCs can be targeted not only in tumor but in sepsis also. Due to the high mortality of sepsis there is an unmet high medical need for novel therapies. It has been published that myeloid-derived cells emerge in septic patients suppressing antigen-driven T cell proliferation, Th1/Th2 cytokine production contributing to higher prevalence of nosocomial infections [76]. Monocytic MDSCs are accumulated in all septic patients whereas granulocytic MDSCs are increased in gram positive cases [77]. It has been published that matured MDSCs lose their inherent immunosuppressive phenotype that could solve the dormant state of the antigen specific immune response in sepsis [78].

However, we have shown that both AML and MDSCs differentiate and dye by apoptosis after the treatment with DU325, although these cells may differ in the underlying molecular mechanisms of differentiation. These molecular pathways need further investigation to reveal. Taken together, our imidazo[1,2-*b*]pyrazole-7-carboxamide derivative is an effective lead compound at nanomolar range to target immature myeloid cells and it is offered for future pre-clinical studies including pharmacodynamics, pharmacokinetics, and safety.

## 4. Materials and Methods

### 4.1. Ethical Statement

The animal experiments were performed in accordance with animal experimentation and ethics guidelines of the EU (2010/63/EU). Experimental protocols were approved by the responsible governmental agency (National Food Chain Safety Office) in possession of an ethical clearance XXIX./128/2013 (2013).

Participant informed consent was obtained prior surgical intervention. The collection of human samples was complied with the Guidelines of the Helsinki Declaration. The isolation of human AML cells was approved by the National Institute of Environmental Health under the 47226-7/2019/EÜIG (2019) ethical license.

The patient AML1 (61-year-old male) was diagnosed with acute myeloid leukemia with cup-like morphology. Laboratory tests showed that bone marrow aspirate cells were in 92% myeloid blasts and CD117^−^/CD34^−^/D13^−^/CD33^bright^/cyMPO+/HLA_DR-/CD38+, 58% positive for CD56 and negative for monocyte markers CD64, CD14, CD300e, and cyFXIII; negative for granulocyte markers CD15, CD16, CD10, and CD11b; and negative for T cell markers CD4, CD7, and CD2. Cytogenetic status was the following, karyotype: normal; FLT3: wild type; CEBPA: wild type; NPM1: insertion (4 nucleotides = nts) in the 12th exon.

The patient AML2 (24-year-old male) was diagnosed with acute myeloid leukemia. Laboratory tests showed that bone marrow aspirate cells were in 85% myeloid blasts, these were in 71% CD117+/CD34+ which cells were CD13+/CD33+/cyMPO+/HLA_DR+/CD38+/CD4^−^/CD11b^−^ and in 84% CD7+, in 14% CD56+, in 14% CD15+, in 12% cyFXIII+. Cytogenetic status was the following, karyotype: normal; FLT3: wild type; CEBPA: duplication (24 nts), insertion (3 nts), point mutation (c.1119A>C), NPM1: wild type.

### 4.2. Cell Culture and Isolation of Human AML Cells

The HL-60 acute promyelocytic leukemia and the mouse mammary carcinoma 4T1 cells were purchased from the American Type Culture Collection (ATCC, Manassas, WV, USA) maintained in Roswell Park Memorial Institute 1640 medium (RPMI-1640) 10% FCS (Gibco, Thermo Fisher Scientific, Waltham, MA, USA) using tissue culture dishes (Corning Life Sciences, Corning, NY, USA). The pH of the cell culture media was controlled to be between 7.2 and 7.4 prior to use. The medium was supplemented with 2 mM GlutaMAX, 100 U/mL penicillin, and 100 μg/mL streptomycin (Life Technologies, Carlsbad, CA, USA) before use. Cells were passed every three days and placed in a humidified incubator at 37 °C 5% CO_2_ (Sanyo, Osaka, Japan).

The aspiration of ten ml bone marrow of AML patients from the pelvis was carried out upon a clinical visit after the signed informed consent of the patients into EDTA Vacutainers (Beckton Dickinson, Franklin Lakes, NJ, USA). Samples were centrifuged 1400 rpm 5 min, the supernatant was removed. Red blood cell lysis was carried out by the incubation of cells with 5 mL ACK (0.155 M NH_4_Cl, 10 mM KHCO_3_, 0.1 mM Na_2_EDTA, pH 7.3, Sigma-Aldrich) solution for 5 min. Samples were loaded on cell strainer (70 μm in pore size) and washed by 20 mL PBS. Cells were counted using Bürker chamber and trypan blue viability dye (Thermo Fisher Scientific) and plated for the treatment as described in the relevant Sections of Materials and Methods.

### 4.3. Isolation of Murine Myeloid-Derived Suppressor Cells

Female Charles River-derivative BALB/c mice (8–10 weeks old) were purchased from Kobay Ltd., (Ankara, Turkey) and were injected orthotopically with 4T1 breast carcinoma cells (1.2 × 10^5^ cells in 100 μL PBS) or sham injected with 100 μL PBS as described previously [29]. The animals had free access to food and water. Biological replicates were analyzed separately from the naive and 4T1 tumor-bearing mice. The weights of the spleens were as follows in the naïve; 99, 8, 13, 14, and 12 mg, and in the tumor-bearing mice; 781, 812, 968, 1350, and 935 m. The weights of the tumors were as follows; 2523, 3037, 2064, 2877, and 1522 mg; the volume of the tumors were as follows; 1272, 1329, 650, 1573, and 807 mm^3^ after 28 days from the injection. The volume of the tumor was measured by a caliper and calculated by the following equation; *D × d^2^ × 0.5*, where *D* = major diameter, *d* = minor diameter as we described previously for the 4T1 breast cancer model [79,80]. After euthanizing the animals, the spleens were removed and homogenized freshly on cell strainer (70 μm pore size, Merck Millipore) using piston of a syringe and sterile PBS. Cells were pelleted by centrifugation 1500 rpm 5 min. The pellet was resuspended in 5 mL ACK lysis buffer for 5 min. Samples were loaded on cell strainer (70 μm in pore size) and washed by 20 mL PBS. Cells were counted using Bürker chamber and trypan blue viability dye (Thermo Fisher Scientific) and plated for resazurin viability assay and immunofluorescent staining upon treatment by imidazo[1,2-*b*]pyrazole-7-carboxamide derivative in RPMI-1640 media supplemented with 10% FCS, 25 μM β-mercaptoethanol, 2 mM GlutaMAX, and 100 U/mL penicillin, 100 μg/mL streptomycin. Cells for flow cytometry were plated into nonadherent Petri dishes (Corning).

### 4.4. Treatment by the Imidazo[1,2-b]pyrazole-7-carboxamide Derivative, DU325

The imidazo[1,2-*b*]pyrazole-7-carboxamide derivative was synthetized at Avidin Ltd. (Szeged, Hungary) as described previously [20]. The compound was DU325, *N*-(4-aminophenyl)-2-(*tert*-butyl)-3-(*tert*-butylamino)-1*H*-imidazo[1,2-*b*]pyrazole-7-carboxamide, the structure is available in the reference [21]. The compound was dissolved in DMSO at 10 mM concentration. Dilutions for the treatment of the cells was prepared in RPMI-1640 complete cell culture media as the concentration is described in the corresponding figure legends.

### 4.5. Resazurin Viability Assay

Cell viability was determined by the fluorescent Resazurin assay as described previously [81]. AML1, MDSCs cells (80,000), were seeded into 96-well plates (Corning Life Sciences) in 80 μL cell culture media. Effect of DU325 was examined in AML1 cells in concentrations 3 μM, 1μM, 333 nM, 111 nM, 37 nM, and 12 nM in 100 μL final volume after 72 h incubation. Effect of DU325 was examined in mouse splenocytes in concentrations 5 μM, 1 μM, 200 nM, 40 nM, and 20 nM in 100 μL final volume after 72 h incubation. Resazurin reagent (Sigma-Aldrich) was dissolved in PBS (pH 7.4) at 0.15 mg/mL concentration, sterile filtered (0.22 μm, Merck Millipore) and aliquoted at −20 °C. We applied resazurin 20 μL stock to 100 μL/well culture. After 12 h incubation at 37 °C 5% CO_2_ (Sanyo), fluorescence (530 nm excitation/580 nm emission) was recorded on a multimode microplate reader (Cytofluor4000, PerSeptive Biosytems). Viability was calculated with relation to untreated control cells and blank wells containing media without cells. The IC_50_ values were calculated by GraphPad Prism^®^ 5.

### 4.6. RealTime-Glo^TM^ MT Cell Viability Assay

The HL-60 cells (2000) were seeded into UV-C sterilized opaque 96-well plates (Tomtec, Budapest, Hungary) in 40 μL media. RealTime-Glo™ reagent (Promega) (2X) was added according to the instructions of the manufacturer. The effect of DU325 was examined in concentrations of 5 μM, 1 μM, 0.2 μM, and 0.04 μM in final volume 100 μL/well. We measured luminescence on a multimode microplate reader (Victor 1430, Wallac, Perkin Elmer) corresponding to live cell number after 1 min to register the baseline and subsequently samples were measured daily.

### 4.7. Z-DEVD-aLuc Caspase Assay

Cells were treated (1 × 10^6^ HL-60 cells in 1 mL media) in 24-well tissue culture plates for 24 h in the concentrations indicated in the graphs and assayed as described previously [82]. Briefly, after 24 h, cells and supernatants were harvested and centrifuged down (3000 rpm, 5 min, 4 °C, Eppendorf). After the withdrawal of the supernatant pellet was lysed in 50 μL 1X Lysis Buffer, stock lysis buffer 5X (250 mM HEPES, pH 7.4, 25 mM CHAPS, 25 mM DTT, Sigma-Aldrich), stored at −20 °C, was diluted prior use freshly in sterile water. After resuspending the pellet, we incubated the samples on ice for 15 min. The debris were pelleted by centrifugation (13,000 rpm, 15 min, 4 °C, Eppendorf). We diluted Z-DEVD-aLuc substrate (10 mM stock in DMSO, Avidin Ltd.) in 1X Assay Buffer, stock Assay Buffer 10X (200 mM HEPES, pH 7.4, 1% CHAPS, 50 mM DTT, 20 mM EDTA) was stored at −20°C and it was diluted prior use freshly in sterile water. The assay was run in 96-well sterile tissue culture plate in a total volume of 100 μL, 5 μL cell lysate was added to 20 μM Z-DEVD-aLuc in 1X Assay Buffer in one well. Blanc samples contained no cell lysate. We incubated the 96-well plate at 37 °C for 1 h. The 50 μL of samples were mixed with 50 μL of Luciferase Detection Reagent (Promega, USA) in a new black wall 96-well microtiter plate. Luminescence was measured by multimode microplate reader (Victor 1430, Wallac, Perkin Elmer) recording counts per seconds (cps). Blanc data were subtracted from all values. Each sample was assayed in triplicates.

### 4.8. LDH Assay

Cells were plated (2 × 10^5^) in 500 μL media in 24-well tissue culture plates and treated as indicated on the graphs. After 72 h cells and supernatants were centrifuged down (3000 rpm, 5 min, at 4 °C). The 50 μL supernatant was measured to one well of the 96-well sterile microtiter plate and the LDH reaction mixture was added (Catalyst solution and Dye solution in 1:45 ratio) in 50 μL according to the instructions of the manufacturer (Roche, Basel, Switzerland). Blanc wells contained media instead of supernatant. After 10 min incubation at RT we measured absorbance at 490 nm by multimode microplate reader (Victor 1430, Wallac, Perkin Elmer). Blanc data were subtracted from all values. Each sample was assayed in triplicates.

### 4.9. Quantitative Real-Time PCR (qRT-PCR)

HL-60 cells (1 × 10^6^) were treated with DU325 in 6-well plates (Corning) in 2 mL in triplicate at 37 °C for 6, 12, 24, and 48 h. Cells were harvested by centrifugation. RNA was purified as described previously [21]. The nucleic acid preparation was done by using the RNA purification kit (Direct-zol^TM^ RNA MiniPrep Kit, Zymo Research, Irvine, CA, USA), following the instructions of the manufacturer. The quality and quantity of the isolated RNA were measured with NanoDrop1000 v. 3.8.1. (Thermo Fisher Scientific, Waltham, MA, USA). Reverse transcription from 3 μg of total RNA was performed with the High-Capacity cDNA Archive Kit (Applied Biosystems, Foster, CA, USA) in a total volume of 30 μL according to the manufacturer’s protocol. After dilution with 130 μL of ultrapure water (Applied Biosystems), cDNA was used as template for gene expression analysis. Quantitative real-time PCR (qRT-PCR) was performed on the LightCycler^®^ 96 System (Roche), using gene-specific primers with SYBR Green protocol, as described previously [83]. Briefly, for cycling, each 10 μL PCR reaction contained 1 μL cDNA (18.75 ng), 250 nM primers, and 5 μL qPCRBIO SyGreen Mix Lo-ROX (2x, PCR Biosystems, London, UK). Primer sequences and accession numbers used in the study are listed in the Table 1. The PCR protocol was as follows; enzyme activation at 95 °C for 2 min, 45 cycles of denaturation at 95 °C for 10 s, annealing at 60 °C, and extension at 60 °C for 10 s. All the PCRs were performed with three replicates. After amplification, the melting curve was checked to verify the specificity of the PCR reactions. The Ct (cycle threshold) values were normalized to ACTB gene for each time point. The presented relative gene expression ratios were calculated using the comparative Ct method (2^−ΔΔCT^). Fold change refers to 2^−ΔΔCT^ treated/2^−ΔΔCT^ untreated. All values were presented as mean ± standard deviation (SD).

### 4.10. Purification of Nuclei and Immunochemical Analysis

Purification of nuclei from HL-60 cells was performed following a previously reported procedure [48]. For Western blot analysis, total lysates from cells and nuclei were separated on 7.5% polyacrylamide denaturing gels and blotted to nitrocellulose membranes (GE Healthcare Life Science, Little Chalfont, UK). The membranes were then reacted with antibodies directed against Vav1, p174-Vav1, and Lamin B (Santa Cruz Biotechnology, Santa Cruz, CA, USA) and against β-tubulin (Sigma-Aldrich, St. Louis, MO, USA), incubated with peroxidase-conjugated secondary antibodies and revealed using the ECL system (PerkinElmer, Boston, MA, USA), as previously reported [84]. The chemiluminescence-derived bands were acquired with ImageQuant™ LAS 4000 biomolecular imager (GE Healthcare), and the densitometrical analysis was performed by means of Image Quant TL software (GE Healthcare), the ratio of phosphorylated Vav (pVav) to Vav1 was quantitated.

### 4.11. Measurement of the MPO Activity

Cells were treated (1 × 10^6^ HL-60 cells in 1 mL media) in 24-well tissue culture plates for 48 h in the concentrations indicated in the graphs. After 48 h cells and supernatants were harvested and centrifuged down (3000× *g*, 5 min, 4 °C, Eppendorf). After the withdrawal of the supernatant, the pellet was fresh-frozen in liquid nitrogen. Samples were freeze-thawed in liquid nitrogen three times, and then centrifuged. A 12 μL aliquot of the supernatant was mixed with 280 μL of phosphate buffer (50 mM, pH 6) containing 0.167 mg/mL of O-dianisidine dihydrochloride and the reaction was started with 10 μL of 0.03% hydrogen peroxide and assayed spectrophotometrically at 490 nm (Benchmark Microplate Reader; Bio-Rad, Budapest, Hungary) after 90 s of shaking [85]. The MPO activity of the samples (U/mL) was normalized to the protein content of each sample. Protein concentration (C) was calculated according to the following equation, C protein (mg/mL) = (1.55 × A280) – (0.76 × A260) × D, where D means the dilution of the lysate. The normalized MPO activity (U/mg) was calculated according to the following equation, enzyme activity (U/mL)/protein concentration (mg/mL).

### 4.12. Fluorescence Flow Cytometry

#### 4.12.1. Detection of the Mitochondrial Membrane Potential (MMP)

Cells (200,000) were plated in 24-well tissue culture plates (Corning Life Sciences) and treated with the indicated concentrations in the figures in 500 μL RPMI-1640 media with 10% FCS for 4 and 24 h. Mitochondrial membrane potential was measured as described previously in reference [21]. After 4 and 24 h, the cells were harvested and centrifuged (2000 rpm, 5 min). The pellet was suspended and incubated for 15 min in 5 μg/mL JC-1 (5,5′,6,6′-tetrachloro-1,1′,3,3′-tetraethyl-imidacarbocyanine iodide, Chemodex, St. Gallen, Switzerland) in final volume 300 μL of cell culture media at 37 °C. Cells (2 × 10^4^) were acquired immediately on a FACSCalibur flow cytometer. Cells were visualized using FL2 (cells with steady state mitochondria) (585/42 nm) and FL1 (cells with depolarized mitochondria) (530/30 nm) channels. Data were analyzed using CellQuestTM software (CellQuest Pro v5.1, Becton Dickinson, Franklin Lakes, NJ, USA). Bar graphs showed the percentage of FL1 positive cells visualized by GraphPad Prism^®^ 5.

#### 4.12.2. Detection of Phosphatidylserine Exposure

Cells (200,000) were plated in 24-well tissue culture plates (Corning Life Sciences) and treated with the indicated concentrations in the figures in 500 μL RPMI-1640 media with 10% FCS. After 48 h incubation for the AML1 cells and 72 h for the murine splenocytes, the supernatants were harvested. Cells were harvested with the corresponding supernatant and centrifuged down (2000 rpm, 5 min, Eppendorf). The pellet was resuspended in Annexin V binding buffer (0.01 M HEPES, 0.14 M NaCl and 2.5 mM CaCl_2_). Annexin V-Alexa Fluor^®^ 488 (Life Technologies, 2.5:100) was added to the cells, which were then kept for 15 min in the dark at room temperature. Before the acquisition, propidium iodide (10 μg/mL, Sigma-Aldrich) was added in Annexin V binding buffer to dilute Annexin V-Alexa Fluor^®^ 488 5X. Cells (20,000 events) were analyzed on a FACSCalibur flow cytometer using CellQuest™ software (Becton Dickinson). The percentage of the FL1 (530/30 nm filter, Annexin V-Alexa Fluor^®^ 488) positive and FL3 (670 nm filter, propidium iodide) negative early apoptotic cells and FL1 positive and FL3 positive late apoptotic cells were determined. The total apoptotic population includes both early and late apoptotic cells. Column charts were created by GraphPad Prism^®^ 5.

#### 4.12.3. Cell Cycle and Sub-G1 Analysis

Cells (200,000) were plated in 24-well tissue culture plates (Corning Life Sciences) in RPMI-1640 10% FCS (Gibco) and were treated with the indicated concentrations in the figures in 500 μL media. After 72 h the supernatant was harvested. Cells were harvested with the corresponding supernatant and centrifuged down (2000 rpm, 5 min, Eppendorf). Pellet was resuspended in DNA binding buffer (1 × PBS, 0.1% tri-sodium-citrate, 10 μg/mL PI, 0.1% Triton X-100, 10 μg/mL RNase A, (Sigma-Aldrich)). After 30 min incubation at room temperature, cells (20,000 events) were acquired on a FACSCalibur flow cytometer (Becton Dickinson), and the sub-G1 apoptotic population was analyzed on FL3 histograms using CellQuest^TM^ software (Becton Dickinson) gating out debris. We gated out doublets for cell cycle analysis which was based on FL2-A/FL2-W dot plots, using ModFit software (v. 2.0, Becton Dickinson). Column charts were created by GraphPad Prism^®^ 5.

#### 4.12.4. Immunofluorescence

Cells (500,000) were plated in 24-well tissue culture plates (Corning Life Sciences) in RPMI-1640 10% FCS (Gibco) and were treated with the indicated concentrations in the figures in 500 μL media. After the incubation time presented in the graph or in the text, supernatant was harvested. Cells were harvested with the corresponding supernatant and centrifuged down (2000 rpm, 5 min, Eppendorf). The antibodies used for flow cytometry are listed in Table 2. Pellet was resuspended and fixed either in 3.5% PBS buffered formaldehyde (Molar Chemicals) for 10 min or resuspended in FACs-buffer (2% FCS, (Gibco) in PBS) for native staining. Cells were washed with FACS-buffer, centrifuged down (2000 rpm, 5 min, Eppendorf). In order to detect pERK, Bcl-xl, pAkt, and active caspase-3, separately, the cells were permeabilized in Permeability buffer (1% FCS, 0.1% saponin (Sigma-Aldrich) in PBS pH 7.4) for 5 min. The detection of CD11b, CD33, CD45, Ly6C, and Ly5G was performed by native cell surface staining gated on PI negative living cells. Cells were washed with FACS buffer, centrifuged down (2000 rpm, 5 min, Eppendorf). After incubation for 1 h at 4 °C with the antibody(s) samples were washed two times with FACS buffer. The secondary antibody detecting caspase-3, anti-rabbit IgG conjugated with Alexa Fluor^®^ 488 (Thermo Fisher Scientific) was diluted to 1:600 and incubated with the cells for 30 min at 4 °C. After washing, 300 μL FACS buffer was added for acquisition with the FACSCalibur flow cytometer and CellQuest^TM^ software (Becton Dickinson) acquiring 20,000 events. The percentage of the positive cells is demonstrated in the graphs. Column charts were created by GraphPad Prism^®^ 5.

### 4.13. Mass Cytometry

Mass cytometry was performed as described previously with minor modifications [29,86]. Briefly, 1.5 × 10^6^ cells were plated in six-well plates for the treatment by 40 nM or 200 nM DU325 or left untreated (untr.) in 2 mL RPMI 10% FCS media for 48 h. After the incubation time, three biological replicates were harvested, pooled, and processed for mass cytometry staining. Cells were centrifuged at 400× *g* for 5 min and assayed for viability by cisplatin staining (5 μM ^195^Pt, Fluidigm San Francisco, CA, USA) for 3 min on ice in 300 μL PBS. Samples were diluted by 1000 μL Maxpar Cell Staining Buffer (MCSB, 201068, Fluidigm) and centrifuged at 400× *g* for 5 min. The pellet was resuspended in 50 μL MCSB and the antibody mix (Table 3) was added in 50 μL at 100X final dilution. The Maxpar^®^ Human Monocyte/Macrophage Phenotyping Panel Kit (201317, Fluidigm,) was used for mass cytometric analysis (Table 3). After 45 min incubation at 4 °C, antibodies were washed two times by 1 mL MCSB and centrifuged at 400× *g* for 5 min. The cells were suspended in the residual volume and fixed in 1.6% formaldehyde (freshly diluted from 16% Pierce formaldehyde with PBS, Thermo Fisher Scientific) and incubated for 10 min at 4 °C. Cells were centrifuged at 800× *g* for 5 min. The DNA intercalator (201192A, Cell ID 191/193 Iridium, Fluidigm) was added in 125 nM in Maxpar Fix and Perm (201067, Fluidigm) in 350 μL for overnight at 4 °C. Next day, the cells for the acquisition were centrifuged at 800× *g* for 5 min then were washed by 2 mL MCSB and centrifuged at 800× *g* for 5 min. Cells were suspended in 1 mL PBS (for WB injector, 107950, Fluidigm) and counted in a Bürker chamber during centrifugation. For acquisition, the concentration of cells was set to 0.5 × 10^6^/mL in cell acquisition solution (CAS, 201241, Fluidigm) containing 10% EQ Calibration Beads (201078, Fluidigm). Cells were filtered through 30 μm gravity filter (CellTrics, Sysmex Gmbh, Bornbach, Germany) and acquired freshly. Mass cytometry data were analyzed in Cytobank (Beckman Coulter, Brea, CA, USA). Viable cells and single cells were determined, viSNE (visualization of stochastic neighbor embedding) analysis (iterations = 1000, perplexity = 30, theta = 0.5), was carried out on 5 × 10^4^ single cell events.

### 4.14. Statistical Analysis

Statistical analysis was performed using two-tailed, homoscedastic Student’s t-test to evaluate the statistical significance (set at * *p* < 0.05, ** *p* < 0.01, *** *p* < 0.001) between two given experimental groups: pairwise comparison of each sample to the untreated control.

## 5. Conclusions

The imidazo[1,2-*b*]pyrazole-7-carboxamide derivative, DU325 drug candidate induces differentiation-coupled apoptosis of immature myeloid cells such as HL-60 and human patient-derived AML cells. The sensitivity of myeloid-derived suppressor cells to DU325 was shown using MDSCs from the murine 4T1 breast cancer model. Pathways, such as ERK1/2 phosphorylation, Bcl-xl induction and Akt phosphorylation, accumulation of Vav1, induction of AP-1 TF complex are involved in the response to DU325 of HL-60 cells. Maturation of HL-60 cells led to apoptotic cell death, the depolarization of mitochondria, activation of caspase-3, and degradation of DNA. The subpopulations of CD7+, CD33+, CD7+/CD33+, CD206+, and CD38^bright^ were the most sensitive to DU325 treatment of human primary AML cells.

## 6. Patents

The imidazo-pyrazole carboxamide derivative of the study has been patented entitled “Imidazo-pyrazole carboxamide derivatives as anticancer agents and the synthesis thereof”, WO2019220155.

## Figures and Tables

**Table 1:** List of the primers used in the study.

Protein Product	Gene Symbol	Accession Number	Forward Primer	Reverse Primer
Myeloid Cell Surface Antigen CD33	CD33	NM_001082618.1 NM_001177608.1	ctgacctgctctgtgtcctg	atgagcaccgaggagtgagt
Jun Proto-Oncogene	JUN	NM_002228.3	ccaaaggatagtgcgatgttt	ctgtccctctccactgcaac
Fos Proto-Oncogene	FOS	NM_005252.3	actaccactcacccgcagac	ccaggtccgtgcagaagt
JunB Proto-Oncogene	JUNB	NM_002229.2	atacacagctacgggatacgg	gctcggtttcaggagtttgt
JunD Proto-Oncogene	JUND	NM_005354.5	cagcgaggagcaggagtt	gagctggttctgcttgtgtaaat
Actin Beta	ACTB	BC002409.2	attggcaatgagcggttc	cgtggatgccacaggact

**Table 2:** List of the antibodies used for flow cytometry.

Target	Host/Clone	Fluorochrome	Supplier, Cat. Number	Dilution in the Assay
pERK1/2 (Thr202/Tyr204)	Mouse mAb, 4B11B69	Alexa Fluor^®^ 488	Biolegend, 675508	1:25
Bcl-xl	Rabbit mAb, 54H6	Alexa Fluor^®^ 488	Cell Sign. Techn. 2767	1:75
pAkt (Ser473)	Rabbit mAb, D9E	Alexa Fluor^®^ 488	Cell Sign. Techn. 4071	1:50
Caspase-3 (Asp175)	Rabbit polyclonal	none	Cell Sign. Techn. 9661	1:500
Anti-rabbit (H+L)	Goat polyclonal	Alexa Fluor^®^ 488	Thermo Fisher Sci. A-11008	1:600
CD11b	Mouse mAb, LT11	FITC	ImmunoTools, 21389113	1:20
CD33	Mouse mAb, WM53	PE-Cy5.5	Thermo Fisher Sci. A15454	1:00
CD45	Mouse mAb, 30-F11	FITC	Biolegend, 103107	1:200
CD11b	Rat mAb, M1/70	PE	Biolegend, 101208	1:80
Ly6C	Rat mAb, HK1.4	APC	Biolegend, 128016	1:80
Ly6G	Rat mAb, 1A8	APC	Biolegend, 127614	1:330

**Table 3:** List of the antibodies used for mass cytometry.

Target	Clone	Metal Tag
CD19	HIB19	142Nd
CD11b	ICRF44	144Nd
CD7	CD7-6B7	147Sm
CD66	CD66a-B1.1	149Sm
CD36	5-271	152Sm
CD163	GHI/61	154Sm
CD45	HI30	156Gd
CD11c	Bu15	159Tb
CD14	M5E2	160Gd
CD16	3G8	165Ho
CD38	HIT2	167Er
CD206	15-2	168Er
CD33	WM53	169Tm
CD3	UCHT1	170Er
HLA-DR	L243	174Yb

## Supplementary Materials

Supplementary materials can be found at https://www.mdpi.com/1422-0067/21/14/5135/s1. The followings are available online. Figure S1: DU325 drives survival pathways, increases pERK, Bcl-xl, and pAkt bright cells, as an early response to treatment, Figure S2: Representative Western blots show the accumulation of Vav1 in whole cell lysates (A) and in isolated nuclei (B) upon treatment, Figure S3: DU325 induces the differentiation, increases the granularity and CD11b expression of HL-60 promyeloblasts, Figure S4: DU325 induces the depolarization of the mitochondrial membrane potential (MMP), Figure S5: DU325 treatment increased the percentage of cleaved caspase-3 positive cells, Figure S6: DU325 treatment increased the percentage of the hypo-diploid apoptotic cells in the sub-G1 population, Figure S7: DU325 hampers the viability of human patient-derived bone marrow aspirate AML cells, Figure S8: Mass cytometric gating strategy of the immunophenotyping of the human patient-derived bone marrow aspirate AML cells upon treatment by DU325, Figure S9: The multiparametric tSNE analysis of human patient-derived AML bone marrow aspirate cells treated by DU325, Figure S10: The CD11b+/Ly6C+ monocytic myeloid-derived suppressor cells are sensitive to DU325 treatment, Figure S11: The CD11b+/Ly6G+ granulocytic myeloid-derived suppressor cells are sensitive to DU325 treatment.
