# Supplementary material for: Imidazo[1,2-b]pyrazole-7-Carboxamide Derivative Induces Differentiation-Coupled Apoptosis of Immature Myeloid Cells Such as Acute Myeloid Leukemia and Myeloid-Derived Suppressor Cells
Source: Int J Mol Sci. 2020 Jul 20;21(14):5135. doi: 10.3390/ijms21145135 (PMC7404197; doi:10.3390/ijms21145135)
Supplement: Supplementary file 1 [file ijms-21-05135-s001.pdf]

International Journal of
Molecular Sciences

MDPI

Supplementary Materials

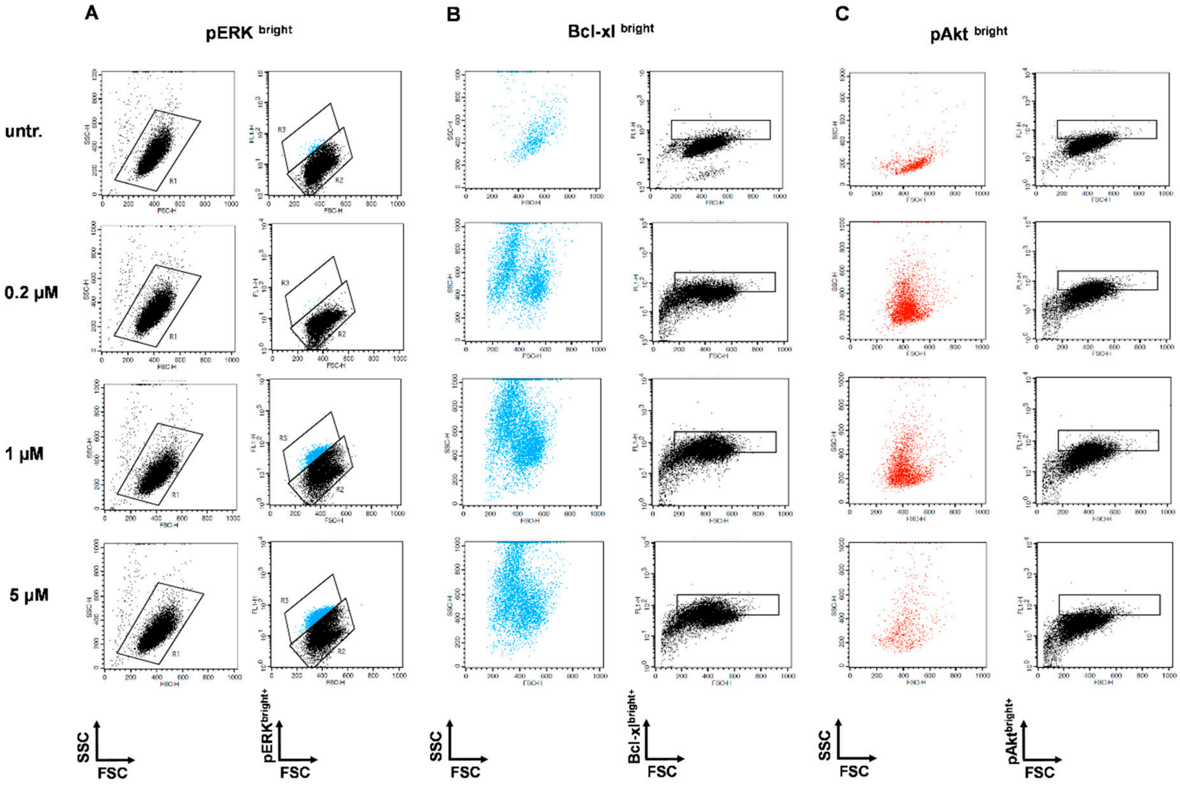

Figure S1. DU325 drives survival pathways, increases pERK, Bcl-xl and pAkt bright cells, as an early response to treatment. Representative dot plots of flow cytometry measurements of pERK ^{bright} (**A**), Bcl-xl ^{bright} (**B**), pAkt ^{bright} (**C**) cells. Cells were treated with 0.2 µM, 1 µM, and 5 µM DU325 for 2 h (**A**) and 24 h (**B-C**). Manual gating was performed to define cells expressing high level (bright) of the proteins under investigation. After 24 h of incubation granularity (SSC = side scatter) of the Bcl-xl ^{bright} (**B**, blue) and pAkt ^{bright} (**C**, red) cells increased. (untr. = untreated; FSC = forward scatter)

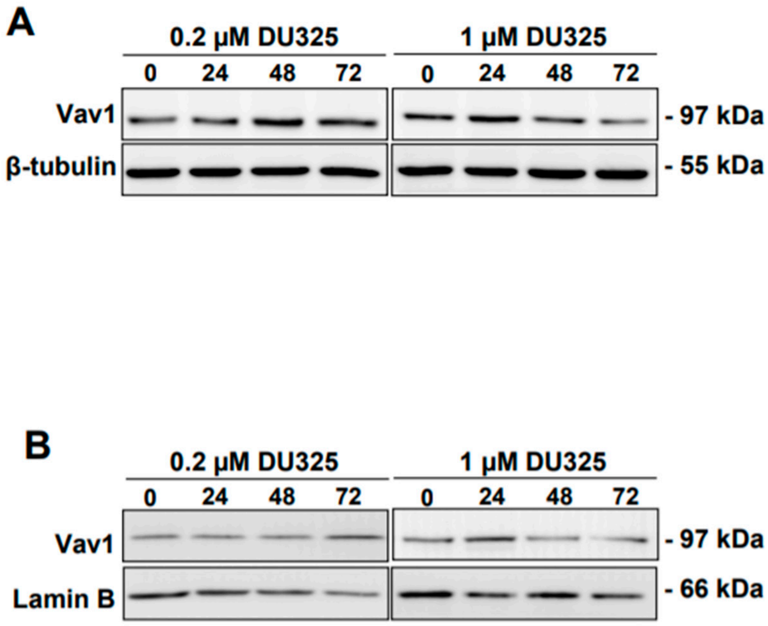

Figure S2. Representative Western blots show the accumulation of Vav1 in whole cell lysates (**A**) and in isolated nuclei (**B**) upon treatment. Western blot analysis was performed with the antibody directed against Vav1 of total lysates from whole cells (**A**) and purified nuclei (**B**) obtained from HL-60 cells cultured in the presence of DU325 for the indicated times (hours). β-Tubulin and Lamin B, respectively, were blotted as internal controls for equivalence of loaded proteins.

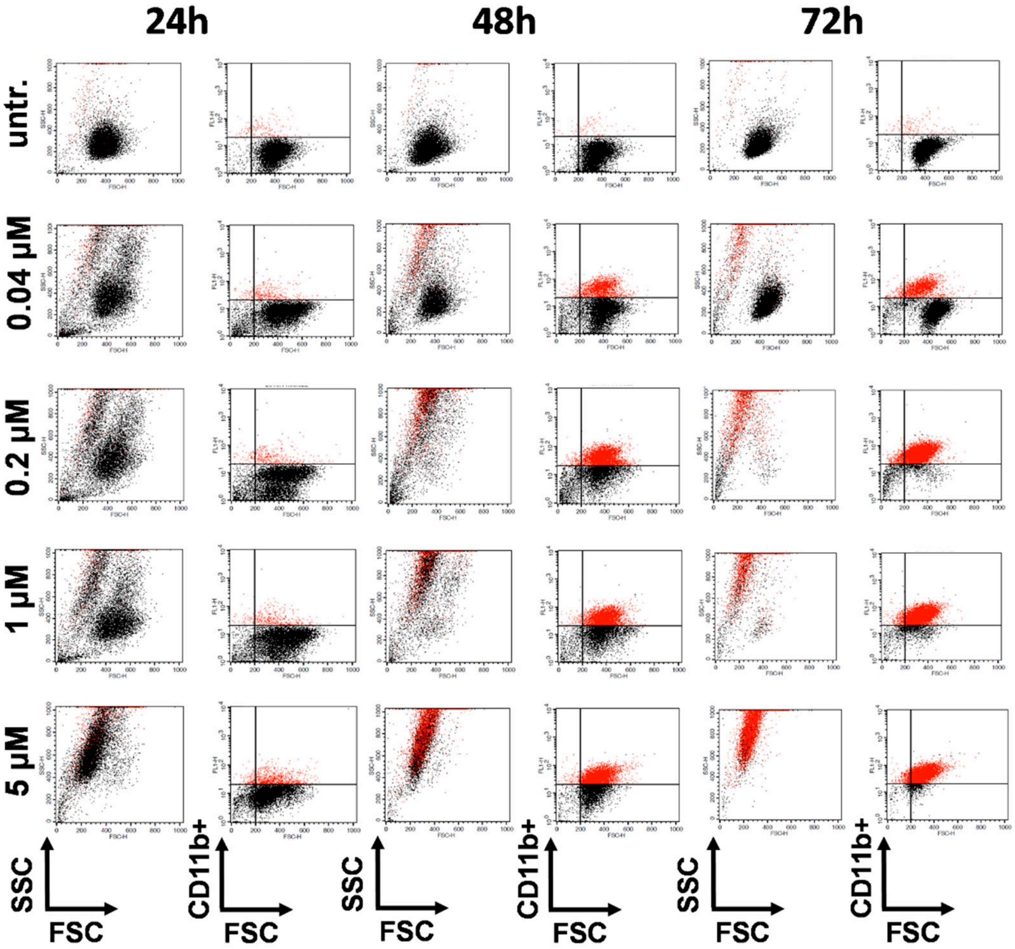

Figure S3. DU325 compound drives the differentiation, increases the granularity and CD11b expression of HL-60 promyeloblasts. Representative dot plots of the CD11b immunostaining of HL-60 cells treated with DU325 (40 nM, 200 nM, 1 µM, and 5 µM) for 24, 48 and 72 h. Both the granularity (SSC) and CD11b expression increased upon treatment.

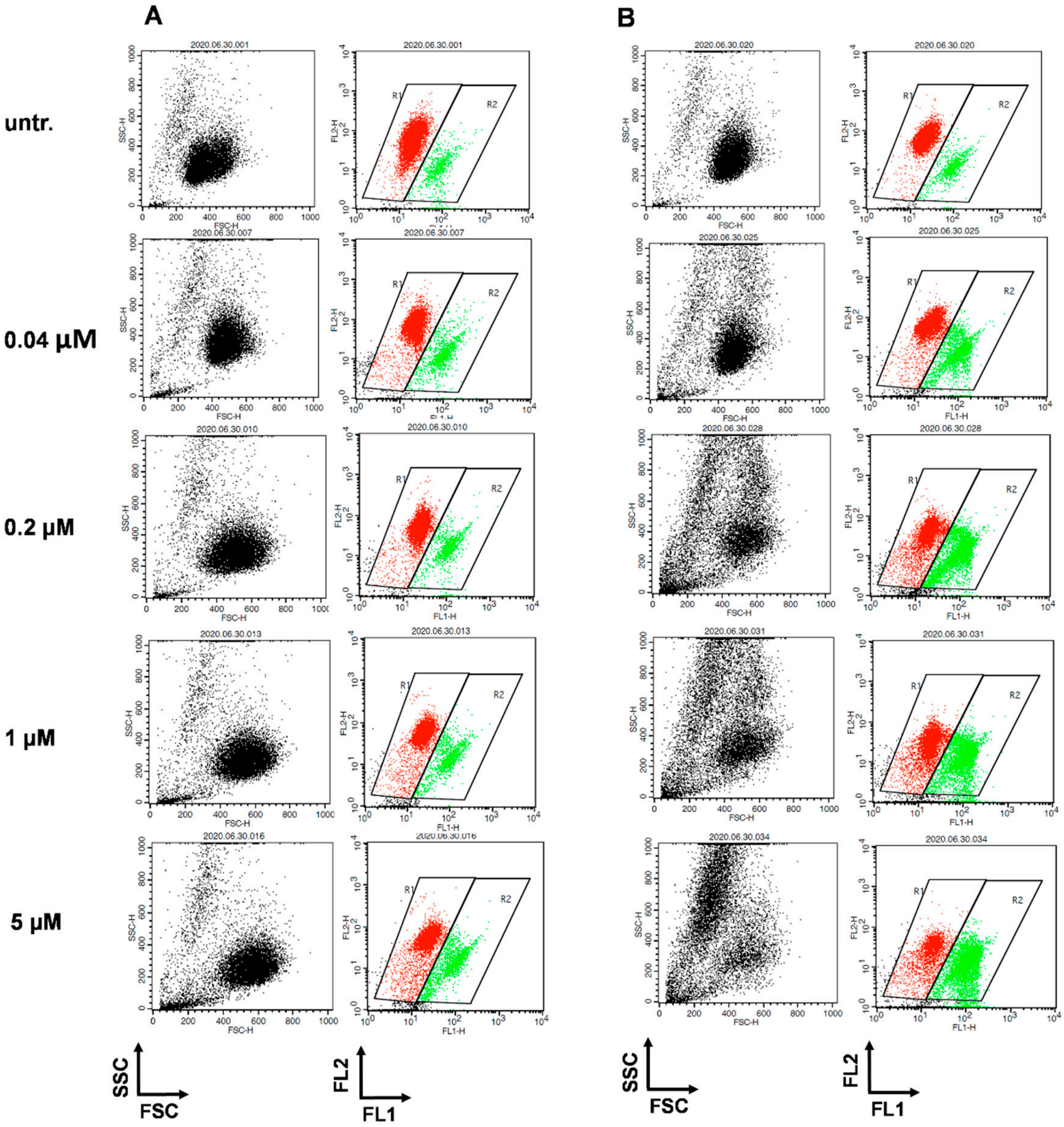

Figure S4. The depolarization of the mitochondria was measured by the JC-1 assay. Cells were treated with 40 nM, 200 nM, 1 µM, and 5 µM DU325 for 4 and 24h. Cells were harvested for the JC-1 staining and acquired by flow cytometry. The JC-1 became green-fluorescent monomer at depolarized membrane potential (FL-1). The percentage of cells with decreased mitochondrial membrane potential (MMP) were quantified.

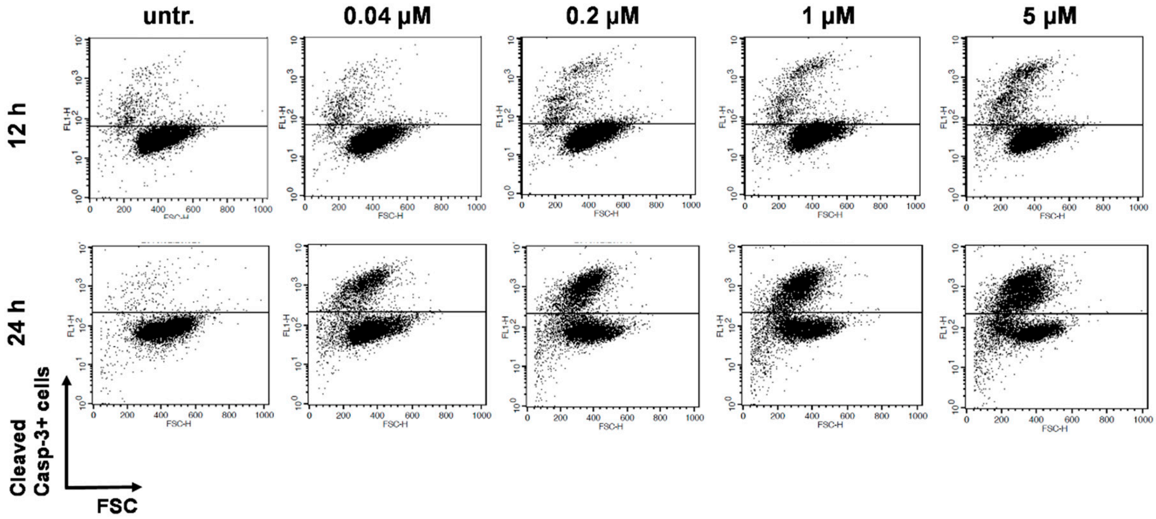

Figure S5. DU325 treatment increased the percentage of cleaved caspase-3 positive cells. Representative dot plots of the increasing percentages of active caspase-3 positive cells after treatment with 40 nM, 200 nM, 1 µM, and 5 µM DU325 for 12 and 24 h.

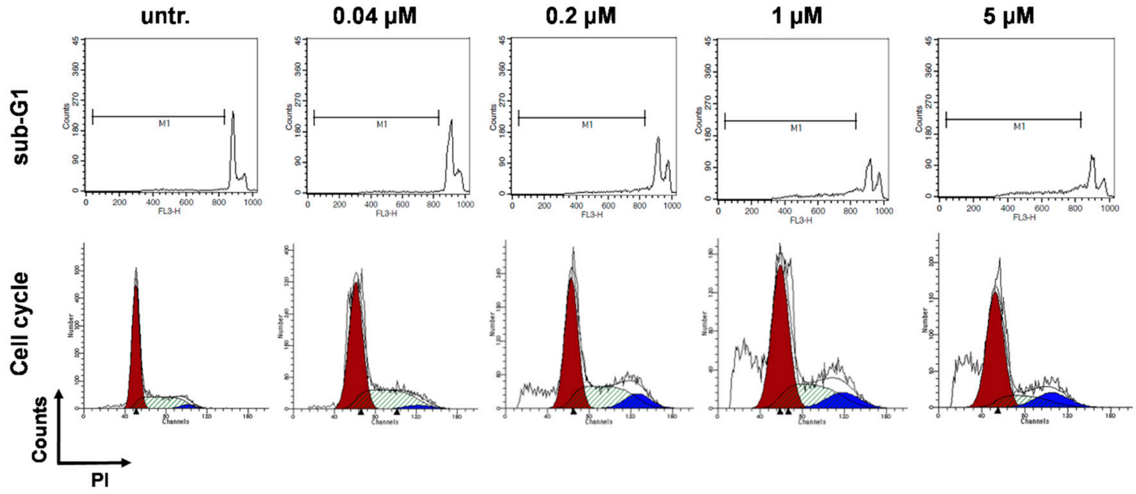

Figure S6. DU325 treatment increased the percentage of the hypo-diploid apoptotic cells in the sub-G1 population. Representative histograms of sub-G1 population and cell cycle phases are shown after treatment with 40 nM, 200 nM, 1 µM, and 5 µM DU325 for 72 h.

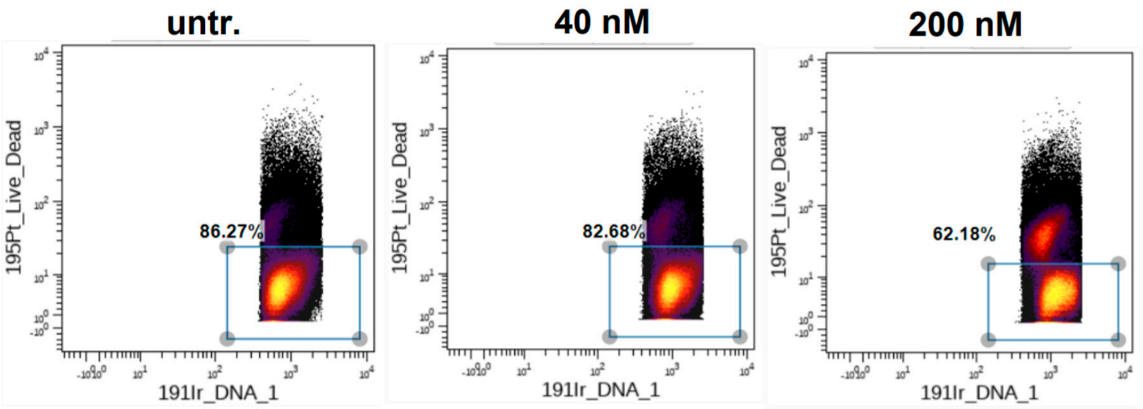

Figure S7. DU325 compound hampers the viability of human patient-derived bone marrow aspirate AML cells. The AML cells of the patent 'AML2' were treated with 40 nM, 200 nM DU325 or left untreated for 48 h. The uptake of Cisplatin ^{195}Pt is proportional with the percentage of cells with decreased viability. Graphs are exported from Cytobank (Backman Coulter).

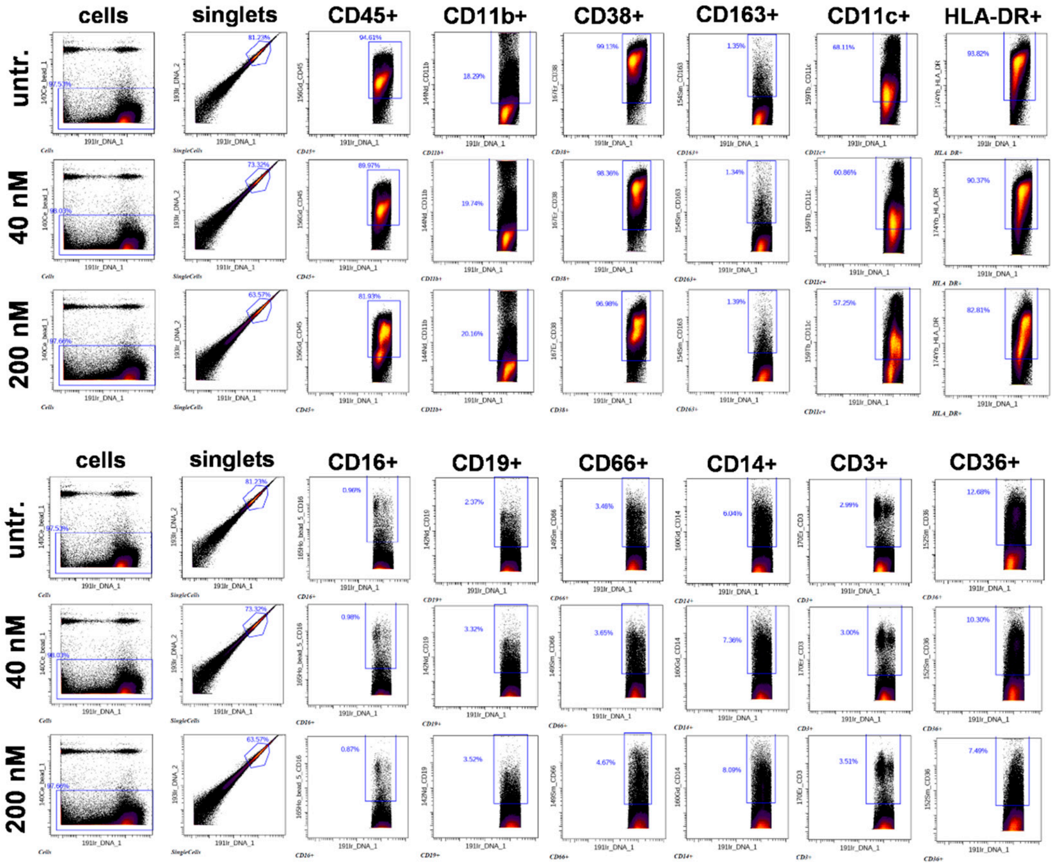

Figure S8. Mass cytometric gating strategy of the immunophenotyping of the human patient-derived bone marrow aspirate AML cells upon treatment with DU325. Dot plots show the manual gating of mass cytometry experiments defining single cells and sub-populations (CD45+, CD11b+, CD38+, CD163+, CD11c+, HLA-DR+, CD16+, CD19+, CD66+, CD14+, CD3+, CD36+) of bone marrow aspirate cells. Graphs are exported from Cytobank (Backman Coulter).

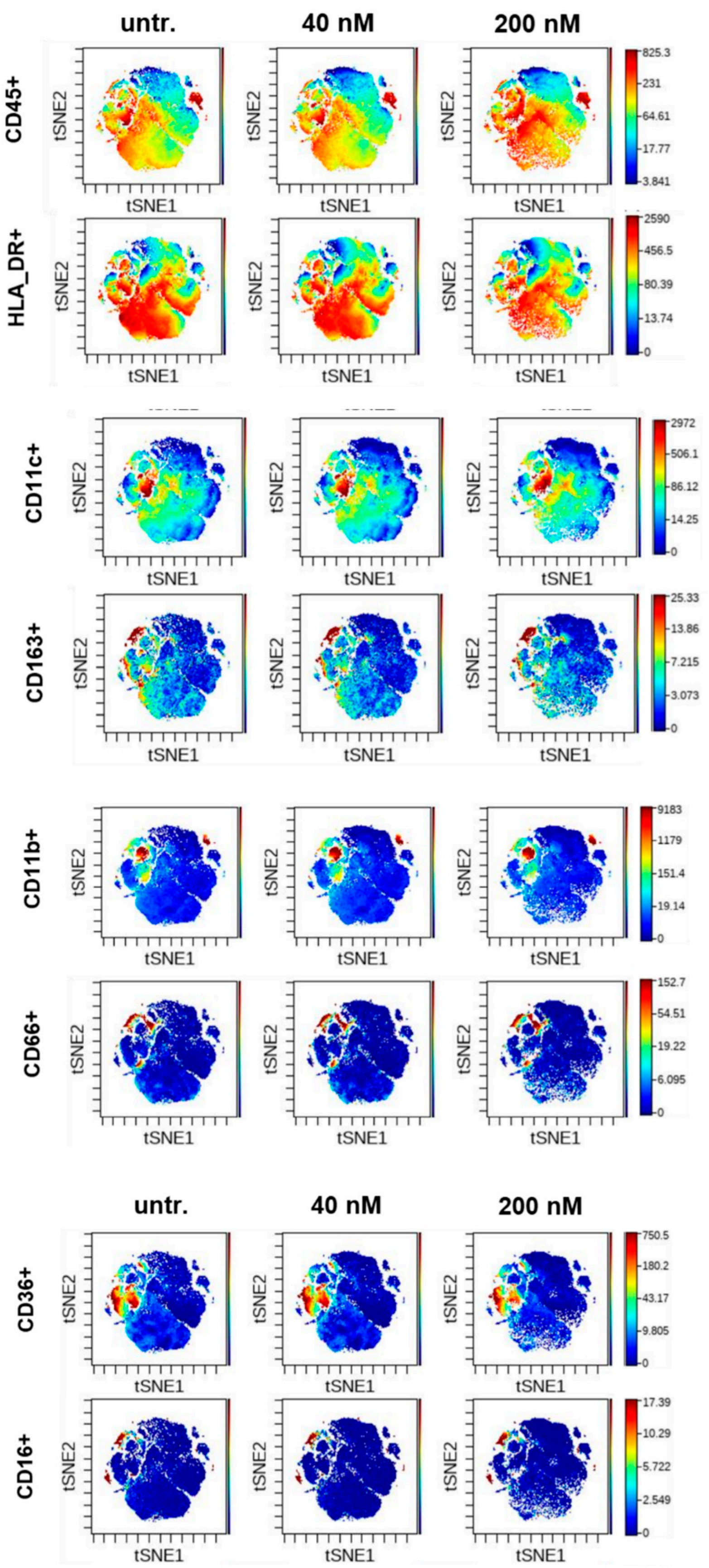
untr.
40 nM
200 nM
CD45+
HLA_DR+
CD11c+
CD163+
CD11b+
CD66+
CD36+
CD16+
tSNE1
tSNE2
825.3
231
64.61
17.77
3.841
2590
456.5
80.39
13.74
0
2972
506.1
86.12
14.25
25.33
13.86
7.215
3.073
9183
1179
151.4
19.14
152.7
54.51
19.22
6.095
750.5
180.2
43.17
9.805
17.39
10.29
5.722
2.549

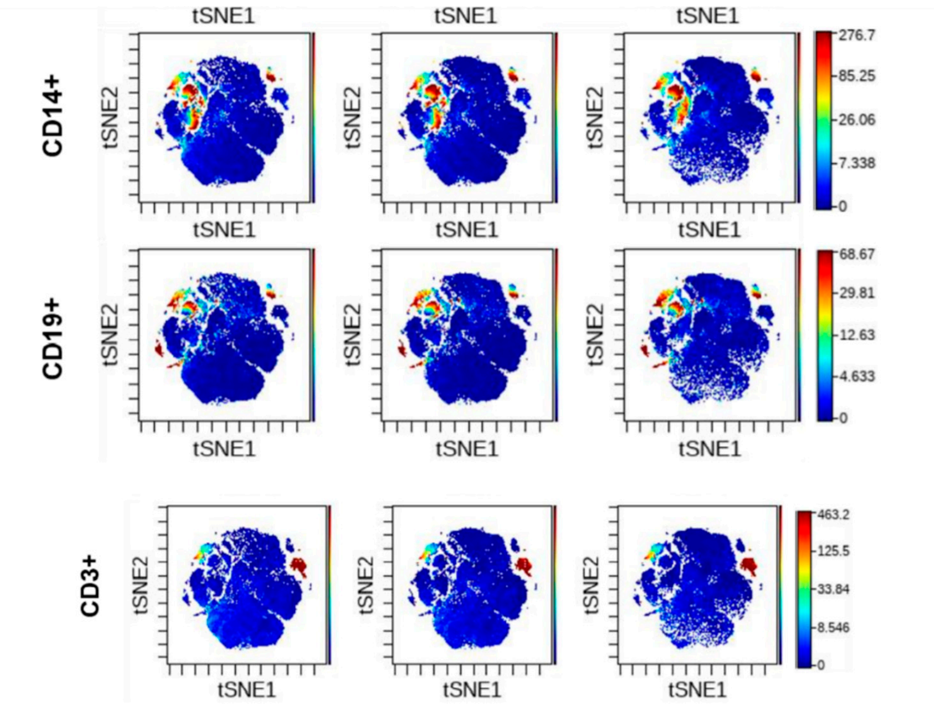

Figure S9. The multi-parametric tSNE analysis of human patient-derived AML bone marrow aspirate cells treated by DU325. The coloration is proportional to the expression intensity (blue = low, red = high). The 'AML2' cells were treated with 40 nM or 200 nM DU325 ex vivo for 48 h. The list of the antibodies used for mass cytometry can be found in Table 3 in Section 4.13. The analysis was performed in Cytobank (Backman Coulter).

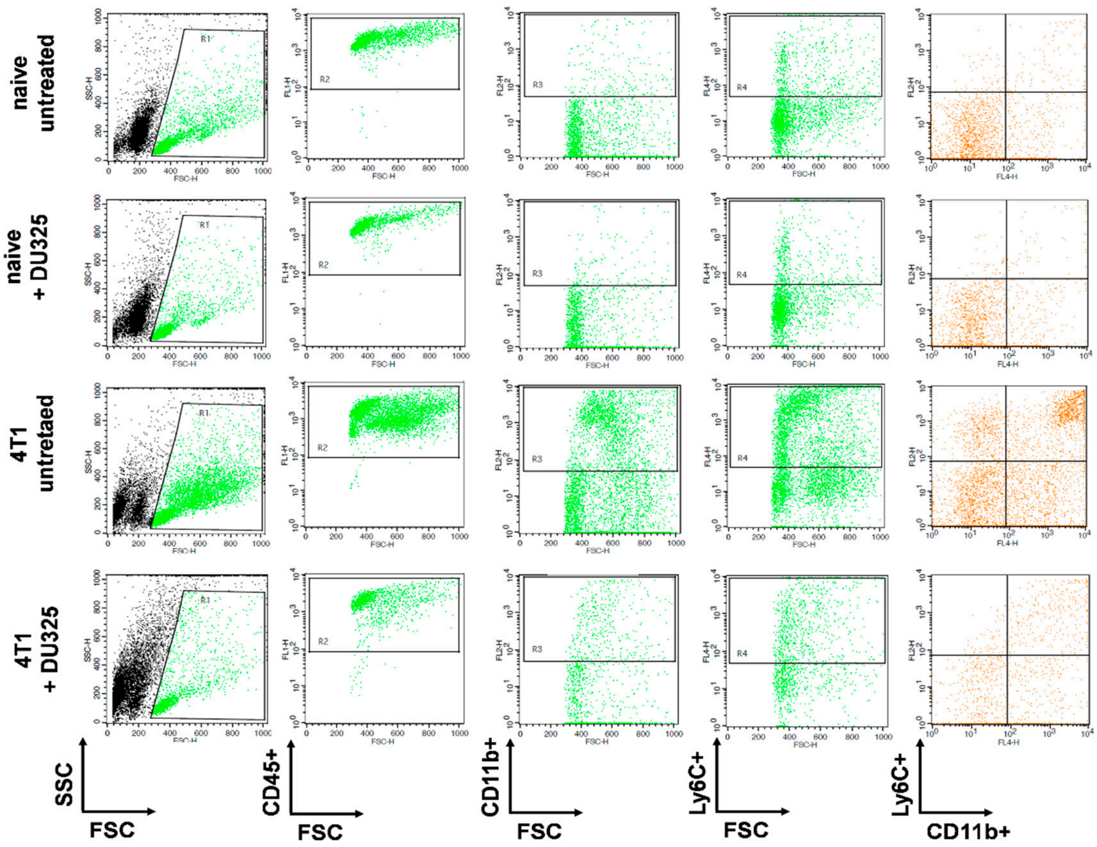

Figure S10. The CD11b+/Ly6C+ monocytic myeloid-derived suppressor cells are sensitive to DU325 treatment. The splenocytes were isolated from 4T1 breast cancer bearing mice and treated with 200 nM DU325 for 72 h. Immunofluorescent staining was performed in order to detect CD11b+/Ly6C+ MDSCs within live (R1 gate in SSC-FSC) and CD45+ cells.

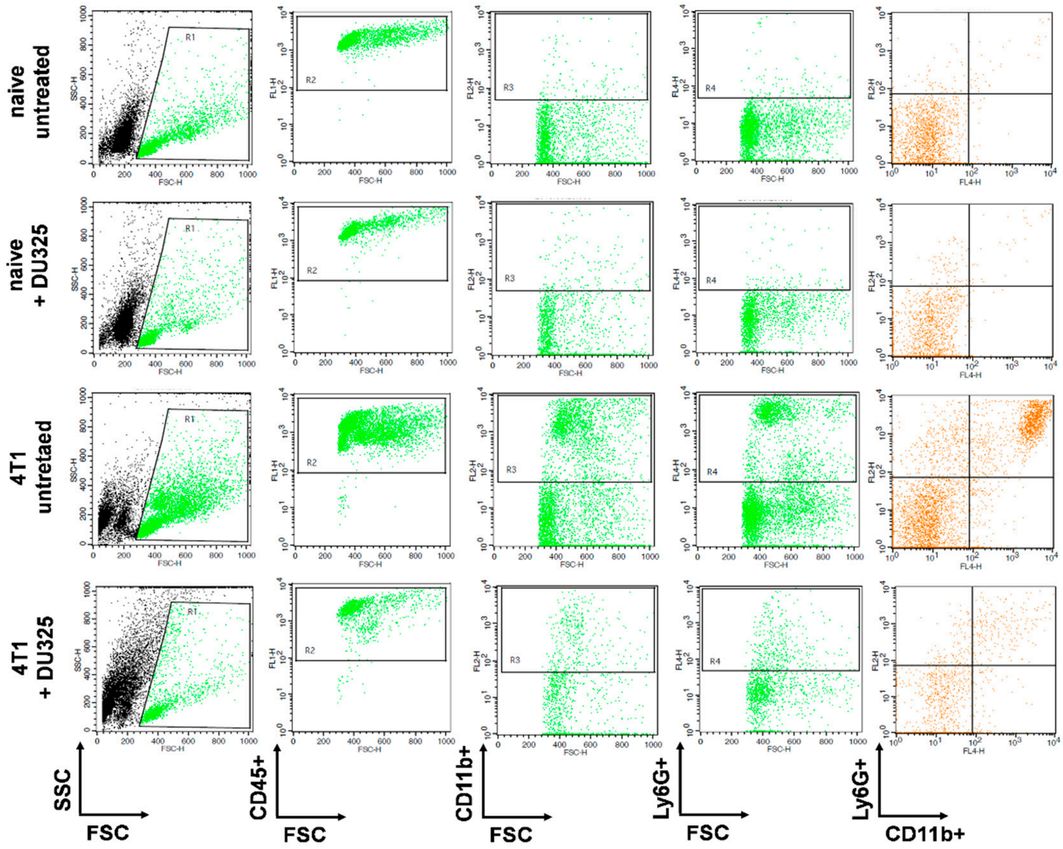

Figure S11. The CD11b+/Ly6G+ granulocytic myeloid-derived suppressor cells are sensitive to DU325 treatment. The splenocytes were isolated from 4T1 breast cancer bearing mice and treated with 200 nM DU325 for 72 h. Immunofluorescent staining was performed in order to detect CD11b+/Ly6G+ MDSCs within live (R1 gate in SSC-FSC) and CD45+ cells.

© 2020 by the authors. Licensee MDPI, Basel, Switzerland. This article is an open access article distributed under the terms and conditions of the Creative Commons Attribution (CC BY) license (http://creativecommons.org/licenses/by/4.0/).
